# The six most essential questions in psychiatric diagnosis: a pluralogue part 1: conceptual and definitional issues in psychiatric diagnosis

**DOI:** 10.1186/1747-5341-7-3

**Published:** 2012-01-13

**Authors:** James Phillips, Allen Frances, Michael A Cerullo, John Chardavoyne, Hannah S Decker, Michael B First, Nassir Ghaemi, Gary Greenberg, Andrew C Hinderliter, Warren A Kinghorn, Steven G LoBello, Elliott B Martin, Aaron L Mishara, Joel Paris, Joseph M Pierre, Ronald W Pies, Harold A Pincus, Douglas Porter, Claire Pouncey, Michael A Schwartz, Thomas Szasz, Jerome C Wakefield, G Scott Waterman, Owen Whooley, Peter Zachar

**Affiliations:** 1 Department of Psychiatry, Yale School of Medicine, 300 George St., Suite 901, New Haven, CT 06511, USA; 2 Department of Psychiatry and Behavioral Sciences, Duke University Medical Center, 508 Fulton St., Durham, NC 27710, USA; 3 Department of Psychiatry and Behavioral Neuroscience, University of Cincinnati College of Medicine, 260 Stetson Street, Suite 3200, Cincinnati, OH 45219, USA; 4 Department of History, University of Houston, 524 Agnes Arnold, Houston, 77204, USA; 5 Department of Psychiatry, Columbia University College of Physicians and Surgeons, Division of Clinical Phenomenology, New York State Psychiatric Institute, 1051 Riverside Drive, New York, NY 10032, USA; 6 Department of Psychiatry, Tufts Medical Center, 800 Washington Street, Boston, MA 02111, USA; 7 Human Relations Counseling Service, 400 Bayonet Street Suite #202, New London, CT 06320, USA; 8 Department of Linguistics, University of Illinois, Urbana-Champaign 4080 Foreign Languages Building, 707 S Mathews Ave, Urbana, IL 61801, USA; 9 Duke Divinity School, Box 90968, Durham, NC 27708, USA; 10 Department of Psychology, Auburn University Montgomery, 7061 Senators Drive, Montgomery, AL 36117, USA; 11 Department of Clinical Psychology, The Chicago School of Professional Psychology, 325 North Wells Street, Chicago IL, 60654, USA; 12 Institute of Community and Family Psychiatry, SMBD-Jewish General Hospital, Department of Psychiatry, McGill University, 4333 cote Ste. Catherine, Montreal H3T1E4 Quebec, Canada; 13 Department of Psychiatry and Biobehavioral Sciences, David Geffen School of Medicine at UCLA, 760 Westwood Plaza, Los Angeles, CA 90095, USA; 14 VA West Los Angeles Healthcare Center, 11301 Wilshire Blvd, Los Angeles, CA 90073, USA; 15 Department of Psychiatry, SUNY Upstate Medical University, 750 East Adams St., #343CWB, Syracuse, NY 13210, USA; 16 Irving Institute for Clinical and Translational Research, Columbia University Medical Center, 630 West 168th Street, New York, NY 10032, USA; 17 New York Presbyterian Hospital, 1051 Riverside Drive, Unit 09, New York, NY 10032, USA; 18 Rand Corporation, 1776 Main St Santa Monica, California 90401, USA; 19 Central City Behavioral Health Center, 2221 Philip Street, New Orleans, LA 70113, USA; 20 Center for Bioethics, University of Pennsylvania, 3401 Market Street, Suite 320 Philadelphia, PA 19104, USA; 21 Department of Psychiatry, Texas AMHSC College of Medicine, 4110 Guadalupe Street, Austin, Texas 78751, USA; 22 Silver School of Social Work, New York University, 1 Washington Square North, New York, NY 10003, USA; 23 Department of Psychiatry, NYU Langone Medical Center, 550 First Ave, New York, NY 10016, USA; 24 Department of Psychiatry, University of Vermont College of Medicine, 89 Beaumont Avenue, Given Courtyard N104, Burlington, Vermont 05405, USA; 25 Institute for Health, Health Care Policy, and Aging Research, Rutgers, the State University of New Jersey, 112 Paterson St., New Brunswick, NJ 08901, USA

## Abstract

In face of the multiple controversies surrounding the DSM process in general and the development of DSM-5 in particular, we have organized a discussion around what we consider six essential questions in further work on the DSM. The six questions involve: 1) the nature of a mental disorder; 2) the definition of mental disorder; 3) the issue of whether, in the current state of psychiatric science, DSM-5 should assume a cautious, conservative posture or an assertive, transformative posture; 4) the role of pragmatic considerations in the construction of DSM-5; 5) the issue of utility of the DSM - whether DSM-III and IV have been designed more for clinicians or researchers, and how this conflict should be dealt with in the new manual; and 6) the possibility and advisability, given all the problems with DSM-III and IV, of designing a different diagnostic system. Part I of this article will take up the first two questions. With the first question, invited commentators express a range of opinion regarding the nature of psychiatric disorders, loosely divided into a realist position that the diagnostic categories represent real diseases that we can accurately name and know with our perceptual abilities, a middle, nominalist position that psychiatric disorders do exist in the real world but that our diagnostic categories are constructs that may or may not accurately represent the disorders out there, and finally a purely constructivist position that the diagnostic categories are simply constructs with no evidence of psychiatric disorders in the real world. The second question again offers a range of opinion as to how we should define a mental or psychiatric disorder, including the possibility that we should not try to formulate a definition. The general introduction, as well as the introductions and conclusions for the specific questions, are written by James Phillips, and the responses to commentaries are written by Allen Frances.

## General Introduction

This article has its own history, which is worth recounting to provide the context of its composition.

As reviewed by Regier and colleagues [1], DSM-5 was in the planning stage since 1999, with a publication date initially planned for 2010 (now rescheduled to 2013). The early work was published as a volume of six white papers, *A Research Agenda for DSM-V *[2] in 2002. In 2006 David Kupfer was appointed Chairman, and Darrel Regier Vice-Chairman, of the DSM-5 Task Force. Other members of the Task Force were appointed in 2007, and members of the various Work Groups in 2008.

From the beginning of the planning process the architects of DSM-5 recognized a number of problems with DSM-III and DSM-IV that warranted attention in the new manual. These problems are now well known and have received much discussion, but I will quote the summary provided by Regier and colleagues:

Over the past 30 years, there has been a continuous testing of multiple hypotheses that are inherent in the *Diagnostic and Statistical Manual of Mental Disorders*, from the third edition (DSM-III) to the fourth (DSM-IV)... The expectation of Robins and Guze was that each clinical syndrome described in the Feighner criteria, RDC, and DSM-III would ultimately be validated by its separation from other disorders, common clinical course, genetic aggregation in families, and further differentiation by future laboratory tests--which would now include anatomical and functional imaging, molecular genetics, pathophysiological variations, and neuropsychological testing. To the original validators Kendler added differential response to treatment, which could include both pharmacological and psychotherapeutic interventions... However, as these criteria have been tested in multiple epidemiological, clinical, and genetic studies through slightly revised DSM-III-R and DSM-IV editions, the lack of clear separation of these syndromes became apparent from the high levels of comorbidity that were reported... In addition, treatment response became less specific as selective serotonin reuptake inhibitors were found to be effective for a wide range of anxiety, mood, and eating disorders and atypical antipsychotics received indications for schizophrenia, bipolar disorder, and treatment-resistant major depression. More recently, it was found that a majority of patients with entry diagnoses of major depression in the Sequenced Treatment Alternatives to Relieve Depression (STAR*D)study had significant anxiety symptoms, and this subgroup had a more severe clinical course and was less responsive to available treatments... Likewise, we have come to understand that we are unlikely to find single gene underpinnings for most mental disorders, which are more likely to have polygenetic vulnerabilities interacting with epigenetic factors (that switch genes on and off) and environmental exposures to produce disorders. [[2], pp. 645-646]

As the work of the DSM-5 Task Force and Work Groups moved forward, a controversy developed that involved Robert Spitzer and Allen Frances, Chairmen respectively of the DSM-III and DSM-IV Task Forces. The controversy began with Spitzer's Letter to the Editor, "DSM-V: Open and Transparent," on July 18, 2008 in *Psychiatric Times *[3], detailing his unsuccessful effort to obtain minutes of the DSM-5 Task Force meetings. In ensuing months Allen Frances joined him in an exchange with members of the Task Force. In a series of articles and blog postings in *Psychiatric Times*, Frances (at times with Spitzer) carried out a sustained critique of the DSM-5 work in which he focused both on issues of transparency and issues of process and content [4-16]. The latter involved the Task Force and Work Group efforts to address the problems of DSM-IV with changes that, in Frances' opinion, were premature and not backed by current scientific evidence. These changes included new diagnoses such as mixed anxiety-depression, an expanded list of addictive disorders, the addition of subthreshold conditions such as Psychosis Risk Syndrome, and overly inclusive criteria sets - all destined, in Frances' judgment, to expand the population of the mentally ill, with the inevitable consequence of increasing the number of false positive diagnoses and the attendant consequence of exposing individuals unnecessarily to potent psychotropic medications. The changes also included extensive dimensional measures to be used with minimal scientific foundation.

Frances pointed out that the NIMH was embarked on a major effort to upgrade the scientific foundation of psychiatric disorders (described below by Michael First), and that pending the results of that research effort in the coming years, we should for now mostly stick with the existing descriptive, categorical system, in full awareness of all its limitations. In brief, he has argued, we are not ready for the "paradigm shift" hoped for in the 2002 *A Research Agenda*.

We should note that as the DSM-5 Work Groups were being developed, the Task Force rejected a proposal in 2008 to add a Conceptual Issues Work Group [17] - well before Spitzer and Frances began their online critiques.

In the course of this debate over DSM-5 I proposed to Allen in early 2010 that we use the pages of the Bulletin of the Association for the Advancement of Philosophy and Psychiatry (of which I am Editor) to expand and bring more voices into the discussion. This led to two issues of the Bulletin in 2010 devoted to conceptual issues in DSM-5 [18,19]. (Vol 17, No 1 of the AAPP Bulletin will be referred to as Bulletin 1, and Vol 17, No 2 will be referred to as Bulletin 2. Both are available at http://alien.dowling.edu/~cperring/aapp/bulletin.htm.) Interest in this topic is reflected in the fact that the second Bulletin issue, with commentaries on Frances' extended response in the first issue, and his responses to the commentaries, reached over 70,000 words.

Also in 2010, as Frances continued his critique through blog postings in *Psychiatric Times*, John Sadler and I began a series of regular, DSM-5 conceptual issues blogs in the same journal [20-33].

With the success of the Bulletin symposium, we approached the editor of PEHM, James Giordano, about using the pages of PEHM to continue the DSM-5 discussion under a different format, and with the goal of reaching a broader audience. The new format would be a series of "essential questions" for DSM-5, commentaries by a series of individuals (some of them commentators from the Bulletin issues, others making a first appearance in this article), and responses to the commentaries by Frances. Such is the origin of this article. (The general introduction, individual introductions, and conclusion are written by this author (JP), the responses by Allen Frances.

For this exercise we have distilled the wide-ranging discussions from the Bulletin issues into six questions, listed below with the format in which they were presented to commentators. (As explained below, the umpire metaphor in Question 1 is taken from Frances' discussion in Bulletin 1.)

1) How to Choose Among the Five Umpires of Epistemology?

Are DSM diagnoses more like constructs or more like diseases? We would like to have the positions of each of the five epistemological umpires stated as clearly as possible.

Umpire 1) There are balls and there are strikes and I call them as they are.

Umpire 2) There are balls and there are strikes and I call them as I see them.

Umpire 3) There are no balls and there are no strikes until I call them.

Umpire 4) There are balls and there are strikes and I call them as I use them.

Umpire 5) Don't call them at all because the game is not fair.

Could you please state the position of the umpire which you endorse?

2) What is a Mental Disorder?

It has been difficult to reach agreement on a definition of mental disorder. Could you comment on this problem, or offer what you think is an adequate definition of the concept, mental disorder?

3) What are the Benefits and Risks of Conservatism?

Given the state of the science of psychiatric disorders, should we design DSM-5 in a conservative manner, with minimal change, or do the state of psychiatric science and the problems in DSM-IV dictate major change?

4) Is Pragmatism Practical?

What roles do science and pragmatism play in the construction of DSM-5? Does our science allow us to make major decisions on a scientific basis? What role do pragmatic considerations play, both when the science is strong and when the science is weak?

5) How Compatible are All the Purposes of DSM?

Is there a conflict over utility in the DSMs? The authors of DSM-III, DSM-IV, and DSM-5 intend the manuals to be useful for both clinicians and researchers. Is there a conflict between what is useful for clinicians and what is useful for researchers? Which group is served better by DSM-III and DSM-IV, and by the prospective changes in DSM-5?

6) Is DSM the Only Way to do Diagnosis?

Given the problems in DSM-III, DSM-IV, and (likely) in DSM-5, would you argue for an alternative, more rational diagnostic system than the DSM? Could you describe it? Would your alternative system simply replace the DSM or restructure it in a major way?

As will become apparent in what follows, these six questions are in multiple ways interrelated, and for that reason a response to one of the questions is often relevant to another of the questions. This is, for instance, quite obvious with Questions 1 and 2. What you think a mental disorder is will affect how you define the notion of mental disorder. Question 4 quickly enters this discussion. Should pragmatic, in addition to purely scientific, considerations enter into your effort to describe and define mental illness? Under Question 1, for instance, Harold Pincus offers a "pragmatic" response that could easily be placed under Question 4.

And now let's bring in Question 3 - whether to take a conservative or activist attitude toward changes in DSM-5. Don't forget that threading its way through all of these questions is the dissatisfaction and disappointment with the scientific status of DSM-III and IV. That troubled status clearly played a role in the epistemological (and ontological) discussion in Question 1, the definitional issue of Question 2, and the pragmatic aspect of Question 4. It is emblematic of the complexity of these discussions that the same troubled state of the current nosology will lead Scott Waterman in an activist direction in Question 3 and Michael Cerullo in a conservative direction.

The final two questions take us in somewhat other directions, but both are related to the discussions that precede them. Question 5, about utility, raises major issues concerning how the manual is actually used, and for whom it is really designed - again, questions related to those of scientific status, definition, pragmatic considerations, and finally attitudes toward change. With this question it's hard to find anyone wanting to defend the premise of DSM-III and IV (and apparently DSM-5) that the manuals are equally useful for clinicians and researchers.

Finally with Question 6 we have an ultimate question - whether the current state of the DSMs warrants a total overhaul. With Ronald Pies we have an individually imagined overhaul; with Joel Paris we have a commentary on DSM-5's effort at revision, and with Michael First's presentation of the NIMH Research Domain Criteria project (RDoC), we have NIMH's response - that the diagnostic manuals of the future may not resemble the DSMs as we know them.

We should not expect from this or any other publication final answers to the questions of psychiatric classification. The questions are too large, and our expectations have to be more modest. What we know is that the goals of DSM-III & IV have not been achieved and that we are left with more immediate questions as to how to proceed with the current revision, DSM-5. Responses to these questions are understandably mixed. What we hope from this article is to keep the discussion going, and perhaps to move it forward a bit.

Finally, because of the total size of this exercise, "The Six Most Essential Questions In Psychiatric Diagnosis: A Pluralogue" will be published in four parts: each of the first three covering two questions and the final part a general conclusion. Thus this article, Part 1, covers the first two questions.

## Question #1: How do we Choose Among the Five Umpires of Epistemology?

*Are DSM diagnoses more like constructs or more like diseases? We would like to have the positions of each of the five epistemological umpires stated as clearly as possible*.

*Umpire 1) There are balls and there are strikes and I call them as they are*.

*Umpire 2) There are balls and there are strikes and I call them as I see them*.

*Umpire 3) There are no balls and there are no strikes until I call them*.

*Umpire 4) There are balls and there are strikes and I call them as I use them*.

*Umpire 5) Don't call them at all because the game is not fair*.

*Could you please the position of the umpire which you endorse?*

### Introduction

Question #1 involves both ontological and epistemological issues: what are psychiatric disorders, and how do we know them? Framing these questions with the metaphor of umpires and balls and strikes comes from Allen Frances's response to commentaries in Bulletin 1, "DSM in Philosophyland: Curiouser and Curiouser." That response offered the positions of three umpires: the realist first umpire, the nominalist second umpire, and the constructionist third umpire. The author sided with Umpire 2, espousing a nominalist stance to the effect that he knows that there is real psychopathology out there but has no guarantee that his diagnostic constructs sort it out correctly. He wrote: "This brings us to me a (call'um as I see'um) second umpire. In preparing DSM-IV, I had no grand illusions of seeing reality straight on or of reconstructing it whole cloth from my own pet theories. I just wanted to get the job done - produce a useful document that would make the fewest possible mistakes, and create the fewest problems for patients" (Bulletin 1, p. 22).

For this article we have added two more umpires: a pragmatist fourth umpire and a fifth umpire who rejects the entire exercise. We were motivated to add these umpires by the fact that some of the responses required them.

Further, we recognize that in asking respondents to choose one position and defend it, we have made an unreasonable demand. Why should an individual not say, I'm a combination of these two umpires, or, I'm a lot of this umpire and a little of that, or finally, I'm a first umpire if we're talking about Huntington's disease, but a second umpire if we're talking about schizoaffective disorder. So, quite understandably, in some our responses we witness the same problem we have with our diagnoses: comorbidity - in this case epistemological (or ontologic) comorbidity rather than diagnostic comorbidity.

In this debate over the nature of psychiatric disorders we experience a tension among the umpires that reflects the status of nosologic science. On the one hand our patients suffer greatly from psychiatric symptoms, and it seems wildly foolish to theorize away their suffering. On the other hand our efforts to organize and classify their suffering can seem arbitrary and confusing. We organize or categorize a symptom cluster and give it a diagnostic name, and it overlaps with another cluster. Or a patient simply has symptoms of both. We start off with the expectation that there will be a match-up between therapeutic agent and diagnostic cluster, and we discover that, at the extreme, most of our pharmacologic agents seem to treat most of our disorders. Finally, we somehow want to resolve this confusion by getting at the underpinnings of the identified disorders, and we discover that the genetics and neuroscience don't support our groupings.

In view of this confusion it's not surprising that opinion divides itself in various ways. Focus on the real suffering out there, along with a conviction that the diagnostic clusters reflect distinct, real conditions, and you end up as a first umpire. Focus on that suffering with uncertainty about the isomorphism between label and disorder, and you become a second umpire. Switch your focus onto the arbitrariness of the labeling, and you end up questioning whether there is anything but the labeling and become a third umpire. Or switch away from the issues of these umpires onto the effects of one label versus another, and you are now a fourth umpire. Finally, decide that it's all nonsense, and you are our fifth umpire.

### Commentary: A Game for Every Kind of Umpire (Almost)

Peter Zachar, Ph.D. and Steven G. Lobello, Ph.D.

Auburn University Montgomery Department of Psychology.

One might think that a philosophical pragmatist should identify with either the pragmatist or the nominalist position in Allen Frances's clever analogy, but that isn't the case. From a pragmatist perspective, philosophical -isms such as realism, pragmatism, nominalism, and constructionism are conceptual distinctions that we make for certain purposes. The question is what information or response options are gained from making these distinctions that would not be gained were other distinctions made.

For example, let's take the pragmatist's view that *I call balls and strikes as I use them*. If taken too literally this is a recipe for a shallow utilitarianism. One of the ethical principles of umpires is to try to make the game as fair as possible - so every batter and pitcher should face the same strike zone (for that umpire). An umpire should attempt to call the pitches as they are (to the best of his ability), and not widen the zone for batters he favors and narrow it for those he does not. Also, in most games, a degree of unreliability in deciding what counts as a ball or strike may not matter, but it can matter a lot in big games. Presumably every psychiatric patient should be treated like a big game, but with 15 minute medication management sessions that is not likely the case. So a kind of realist attitude is important for keeping the game fair. This is true of psychiatric nosology as well. We should always attempt to classify the world as it is not how we want it to be. A pragmatist would not deny the spirit of this ethic.

Most pragmatists would point out that the purpose of the strike zone is to assure that the batter has a chance to hit the ball well enough to get on base. He cannot do so if the pitch is too high, in the dirt, or wide of the plate. This makes the strike zone a practical kind. There are also practical constraints on the strike zone's location that create a kind of objectivity - but beyond that there is no gold standard. Furthermore, it is not true that every pitch that goes through the zone on the way to the catcher is a strike. For example, spit balls have such unpredictable trajectories that batters have very little chance of hitting them, and they are therefore illegal whether or not they are in the zone. Psychiatry lacks fixed gold standards as well, and the social implications of giving a diagnosis that is contrary to the purpose of diagnosing can also affect whether something is considered to be an official disorder (e.g., pedophilia).

What of the nominalists who say *I call balls and strikes as I see them*? Perhaps a better way to think about nominalists is that they deny both that the criteria for balls and strikes were created by the Platonic baseball gods and that competent umpires can recognize what is naturally a ball and naturally a strike. Cousins to the pragmatist, the nominalists say that what exist are particular pitches, and we tend to group them into the ball category or the strike category for various and sundry reasons. Very different pitches like fast balls, curve balls and sliders can all be strikes. These groupings can also be altered. For example up until the 1920s the spit ball was a legitimate pitch (as homosexuality was once considered a legitimate psychiatric disorder).

So nominalists and pragmatists are uncomfortable when realists start talking about fixed world structures and natural kinds. There are, however, *kinds *- fastballs, curve balls, etc. With the realists, the pragmatists and nominalists recognize the value of understanding the causal mechanisms that produce these kinds (e.g., Vaseline helps you throw good spit balls), but individual pitches can be grouped in a plurality of ways.

The constructionist position is the easiest to defend in this example because baseball is a social construction, and like other social constructions such as the U. S. Government and currency, baseball is a real thing. So what information do we gain from the constructionist analysis? Rather than saying *There are no balls and strikes until "I" call them*, it is more accurate to say that social construction is a historical and community activity. Baseball proper did not exist in 1800 and a pretty good story can be told about the social and economic factors that helped shape the game we have today. A similar narrative could be developed for psychiatry, for example, there is a good story to be told about how degeneration theory in the 19^th ^century and pharmaceutical marketing practices in the 20^th ^century both shaped the classification system. Social constructionists would also point out that something like the introduction of the designated hitter was not a deductive consequence of the rules of the game. Its legitimacy has to be understood with respect to the baseball community and its chosen authorities. Something similar is true of the scientific community and its designated authorities, including the process by which the DSM and the ICD is developed. The pragmatists consider this useful information.

Finally we come to the Szasian. It is a category mistake to lump a political and ethical position such as *I refuse to play because the game is not fair *with realism, pragmatism, nominalism and constructionism. Anti-psychiatry is better considered a behavioral option available to a disillusioned realist. In terms of baseball, the claim would be that in the rest of sports, things like field goals and holes-in-one are objectively fixed, but there is so much variation between umpires in terms of the strike zone, that any rational person would see that the so-called objectivity of the game is a myth. Other like-minded critics would point out that there seems to be statistical evidence that the strike zone gets wider when the count is full - which keeps the game exciting. It is also economically convenient for the sport as a whole if pitchers are allowed some leeway when being close to throwing perfect games and batters allowed leeway when being close to breaking hitting records. Field goals and holes-in-one do not work like that, say the critics, yet baseball wants its consumers to think it is like those other sports. Perhaps the best argument against the Szaszian view is to point out that if they studied football and golf more closely, they might see that things are not as always as objective over there as they assume. Baseball should not be evaluated with respect to an idealized image of other sports just as psychiatry should not be evaluated with respect to an idealized image of other medical specialties.

### Commentary: Mental Disorders, Like Diseases, Are Constructs. So What?

Claire Pouncey, M.D., Ph.D.

Philadelphia, PA.

The literature on the philosophy of psychiatric nosology often conflates questions of ontology - i.e., whether mental disorders exist as abstract entities- with questions of epistemology - i.e., how we can know anything about them if they do. To ask whether mental disorders are (actual) diseases or (mere) constructs confuses these two types of questions about mental disorders, as I will use the first three umpire positions to illustrate. This error is prevalent in academic discussions about psychiatric nosology.

Ontologic commitments are basic metaphysical commitments about what exists in the world. Most of us, by virtue of the fact that we operate in our physical and social worlds as we do, are committed to the existence of intersubjectively appreciable mid-level objects, such as plants, buildings, bodies of water, and other persons, to name just a few. That is, we are realists *about *(and realism is always local to a particular question) mid-level objects, as evidenced by our behaviors.

It is easier to be skeptical (a.k.a. antirealist) about invisible, microscopic, macroscopic, and abstract objects. Most of us are ontologically committed to the existence of oxygen, given what we know about basic physiology and the chemistry of our natural environment, although it is microscopic in its elemental form and undetectable by the senses in its macroscopic form. Our commitments to microscopic entities such as muons, macroscopic entities such as red giants, intangible phenomena such as global warming, or second-order (categorical) entities such as phyla may be much weaker, and more prone to debate.

Mental disorders generate ontological skepticism on several levels. First, they are abstract entities that cannot be directly appreciated with the human senses, even indirectly, as we might with macro- or microscopic objects. Second, they are not clearly natural processes whose detection is untarnished by human interpretation, or the imposition of values. Third, it is unclear whether mental disorders should be conceived as abstractions that exist in the world apart from the individual persons who experience them, and thus instantiate them. Together, these reasons to doubt the ontic status of mental disorders become quite persuasive.

Setting ontological antirealism aside, we can ask epistemological questions separately: if we assume that mental disorders do exist as abstract entities, how do we go about studying them, and on what basis can we possibly gain genuine knowledge about them? Even if we collectively agree that, for example, a particular person at a given time were experiencing a major depressive episode, on what grounds can we know that 'major depressive disorder' exists as an abstract entity? On what grounds can we infer that the broader class 'mood disorders', or 'mental disorders' as the most general class, exist as further abstractions? Epistemic realists may be realists about *Hector's *depression, about the existence of an abstract entity that is major depressive disorder, or about the existence of mental disorders in the world generally. They may not be realists about all three. Similarly, epistemic antirealists may doubt one or more of these commitments.

Umpire #1 is both an ontological realist and an epistemological realist about balls and strikes in baseball. Balls and strikes are real things (events) that exist (happen) in the world, and Umpire 1 has the means and ability to detect them in accurate and unbiased ways: "There are balls and there are strikes and I call them as they are." This tends to be the position attributed to psychiatry. Psychiatry's rhetoric, if not the actual commitments of all practitioners, says both that mental disorders are abstract entities that exist in the world and manifest in individual persons, and that these processes can be intersubjectively appreciated and elucidated as they truly are. Let's call this the Strong Realist position.

Such confidence is not exhibited by Umpire #2, who shares the ontological realism of Umpire #1, but not the epistemological realism. In tempering his epistemological position to "I call them as I see them," Umpire #2 maintains that balls and strikes exist apart from his perception of them, but softens his position to recognize that he may not always perceive them as they exist in the world. That is, Umpire #2 is *ontologically *committed to the existence of balls and strikes, but does not assume that he always has epistemic access to that reality. Let's call this the Strong Realist/Weak Constructivist position.

Umpire #3 is an ontological and an epistemological antirealist about balls and strikes: no balls or strikes exist in the world regardless of who thinks they might. In calling them, the umpire constructs the truth. This is not necessarily to say that all his calls are unfounded fictions, but rather it is to say that although the umpire describes his perceptions as accurately he can, there is no ultimate, underlying reality to which those perceptions could be compared, even in the absence of epistemic limitations. Let's call this the Strong Constructivist position.

Psychiatry's strongest critics tend to make strong constructivist arguments: mental disorders do not exist, so any diagnosis, treatment intervention, or research finding is exempt from ultimate confirmation or refutation. In their strongest form, calling mental disorders 'constructs' *is *meant to communicate that they are mere fictions, completely unfounded medical lore. However, note that on the Strong Realist/Weak Constructivist view this is not the case. Calling a mental disorder a 'construct' does not imply invention so much as it serves as a reminder that our epistemic access to the reality of things is always limited. On this view, every abstract entity is a construct, and constructs can be legitimate objects of scientific investigation. Often, there is broad agreement about the nature of scientific constructs, such as phyla, subatomic particles, or diseases, even if the construct is construed as a working hypothesis, or a category of disparate entities that does not lend itself to simple definition or characterization. On this view, mental disorders are like diseases: they are a heterogeneous class of abstract entities that have uncertain ontic status apart from the persons who instantiate them. In formalizing its nosology, psychiatry is trying to call them as we see them.

### Commentary: Why Umpires Don't Matter

Nassir Ghaemi, M.D.

Tufts University Department of Psychiatry.

Nietzsche said truth is a mobile army of metaphors. If you get your metaphor wrong, you'll miss the truth. I think this is the case with the umpire metaphor that seems to be the central concept underlying the thinking of my interlocutor. I think it is simply wrong-headed. It sets up psychiatry and science and knowledge as a game, where the rules can be changed, and where there may be no truth. If you are a postmodernist extremist, this may make sense. But if you accept that there are truths in the world (such as that if you take very high doses of lithium, you will get toxicity), then it makes no sense.

A mistaken metaphor has no response except to say that it is mistaken.

Before offering a better metaphor, let me say that I accept the realist position, that is, that diseases exist independent of me and you that are expressed as psychiatric symptoms like the chronic delusions of schizophrenia, or the mood states of manic-depression. To prove this fact, I suggest three approaches. One, suggested by Paul McHugh, is to actually see people who have these symptoms, the old kick the table test of realism. The second is to debate the merits of the positions pro and con; I won't do so here, but I think others have done so in reasonably persuasive ways, such as Roth and Kroll's *Reality of Mental Illness*. The third is to apply the pragmatic test, and see the consequences of one position or the other. I accept the realist view in at least some psychiatric diseases, but I would add that if one does not, he or she should think of the consequences. I don't see how one can reject the reality of psychiatric disease, and still practice psychiatry, especially with the use of harmful drugs.

This metaphor brings out those stark choices, as well as provides further rationale for the reality of at least some psychiatric diseases based on how matters have gone in other examples of similar problems in the history of science and medicine.

Here then is a better metaphor for understanding psychiatric nosology, one that I heard from Kenneth Kendler and which I am expanding here. In a presentation on "epistemic iteration," building on work in history of science, Kendler described how we can understand any scientific process as involving an approximation of reality through successive stages of knowledge. The main alternative to this process is "random walk" where there is no trend toward any goal in the process of scientific research. Kendler points out that epistemic iteration won't work if there are no real psychiatric illnesses. If these are all, completely and purely, nothing but social constructions, figments of our cultural imaginations, then there is no point to scientific research at all. (I would add: to be honest doctors, we should stop thereby killing patients with our toxic drugs - since all drugs are toxic - stop taking their money to buy our large houses, and retire.) The random walk model is a dead end for any ethical practice of medicine, because if there is no truth to the matter, then we should not claim to have any special knowledge about the truth.

If there is a reality to any psychiatric illness, then epistemic iteration makes sense, and indeed it has been the process by which much scientific knowledge has been obtained in the past. Take temperature. A long process evolved before we arrived at the expansion of mercury as a good way to measure temperature. There was a reality: there is such a thing as hot and cold temperatures. How we measured that reality varied over time, and we gradually have evolved at a very good way of measuring it. Temperature is not the same thing as mercury expansion: our truth here is not some kind of mystical absolute knowledge. But it is a true knowledge.

A similar rationale may apply to psychiatric diseases. We may, over time, approximate what they are, with our tools of knowledge, if we try to do so in a successive and honest manner, seeking to really know the truth, rather than presuming it does not exist.

The better metaphor, then, which captures epistemic iteration versus random walk alternatives would be to think of a surface, and a spot on that surface, which we can label X, representing the true place we want our disease definition (see figure). If we were God, we would know that X is the right way to describe the disease. Let A be our current knowledge. How do we get from A to X. One way is to go from A to B, from B to C, from C to D, in a zig zag pattern, as our research takes us in different directions, but gradually and successively closer to X. This is epistemic iteration.

The random walk pattern would involve the same starting point A, and multiple movements to B, C, and D, but with no endpoint, because no X would exist (see figure). In this process, movement is random, there is no reality pulling scientific research towards it, like gravity pulling objects closer, and there is no end, and no truth. If this is the nature of things, then our profession has to admit to everyone everywhere that this is what we are doing. We should then give up any claims to specific knowledge and stop treating - and harming - people.

**Figure 1:**
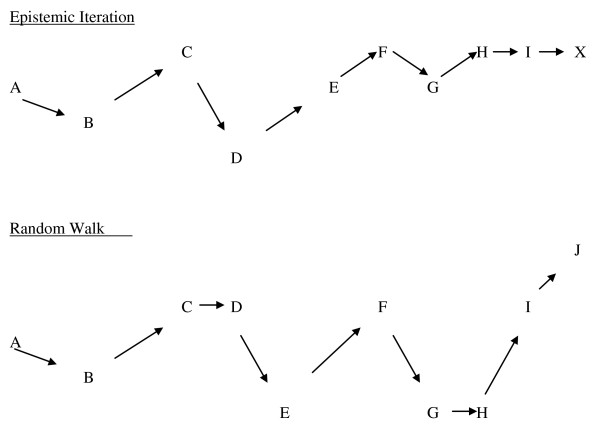
**Epistemic Iteration Versus Random Walk**.

The history of medicine and the history of science gives many examples of both approaches. So the question really is an ontological one: do mental illnesses exist as realities in the external world, as biological diseases independent of our social constructs and personal beliefs? The umpire metaphor assumes, but does not answer, that question. The epistemic iteration metaphor shows how the answer to that question faces us with two opposed choices about how we understand science and psychiatry. If psychiatry is like the rest of medicine, if there are some psychiatric diseases that are independent biological realities just as there are some medical diseases, then the epistemic iteration metaphor would seem valid in some cases, and the umpire metaphor, useless as it is, should be discarded. Figure 1.

### Commentary: The Three Umpires of Metaphysics

Michael Cerullo, M.D.

University of Cincinatti Department of Psychiatry.

The debate about the nature of the external world has been a central question of metaphysics since the first pre-Socratic philosophers. Most working scientists and philosophers today would be classified as modern realists who believe there is an independent objective external reality. Within the realist camp there is further debate about just how much we can know about absolute reality. Immanuel Kant termed the underlying reality of the world "the thing in itself" (*das Ding an sich*) and believed we could never truly know this ultimate reality [34]. Opposed to the realists are the anti-realists who hold that there is no independent objective reality separate from our own subjective experience. Allen Frances' umpire analogy is a good way to frame the major positions in this debate [2](Francis 2, 21-25). Frances' first umpire who believes there are balls and strikes and calls them as they are is a modern realist. Umpire two is a Kantian realist who believes there are balls and strikes but can only call them as she sees them. Umpire three is an anti-realist who believes there are no balls and strikes until he calls them.

These days it is hard to seriously defend an anti-realist position in science. Neuroscientists contend that all behavior, from depression to extroversion to a dislike of tomatoes, is dependent and explainable by the workings of the brain. On the other hand there is still a real debate as to whether subatomic particles are the final bedrock of reality or a mere appearance of a deeper reality (strings? more particles all the way down?). However this latter Kantian uncertainty doesn't seem to have much relevance to the debate about the brain. After all, it doesn't seem to make any difference in our understanding of neurons if their atoms are ultimately made of strings or point particles.

Outside of metaphysics there is another parallel to the umpire analogy in epistemology. Within epistemology there is a subfield interested in the taxonomy of illness. The two major groups in this debate are the naturalists and normativists [35,36]. Naturalists believe disease can be defined objectively as a breakdown in normal biology. The naturalist position corresponds to the first umpire. Normativists believe our definitions of disease are subjective and culturally driven and thus identify with the third umpire. The second umpire seems to mix elements of both epistemological positions.

My own sympathies lie with modern realism when it comes to behavior and a combination of normativist and naturalist positions when defining disease. Although there is physical explanation for all behavior (hence my realist position), not everything in the universe is physical. Definitions of disease require value judgments, and while each value judgment surely has a physical explanation in the brain, nothing physical can decide which judgment is correct. Even in areas of medicine outside psychiatry there is often a strong normativist element in how diseases are defined. Many diseases such as hypertension or hypercholesterolemia require making arbitrary cut off points in laboratory values. Deciding these cut off points requires making hard decisions about public health and considering the risk/benefit ratio of any decision. There is clearly a strong normativist element in theses definitions, yet clearly that does not make them bad or incorrect descriptions. Many psychiatric diseases also have a similar logic. While everyone has some sad mood or anxiety there are obvious extremes which are justifiably labeled as mood or anxiety disorders. Once again there may be certain arbitrary cut off points when deciding how much sadness or anxiety is too much but that does not invalidate these definitions anymore so that it would the "physical" illness listed above. This being said, there are also many diseases that are much better defined from a more naturalist perspective. For example, in psychiatry schizophrenia seems to be better defined from the naturalist perspective along with other physical diseases like Parkinson's disease or dementia. It seems easier to define these diseases using the naturalist ideal of disease as a breakdown in the "typical" human biology.

The lesson in these debates is that psychiatrists (and the public) should recognize that all definitions of disease have normativist and naturalist elements even in a world described by a scientific realism. None of Frances' umpires fits with my combined metaphysical and epistemological positions. Therefore I suggest a different umpire, one who believes in an objective physical world that we can access to determine exactly what are balls and strikes. Yet it is the umpire and players who first must choose the rules of the game, some of which may always seem arbitrary but the majority of which are constrained by the physics of balls and bats and the semantic and historical notions of games and baseball.

### Commentary

Jerome C. Wakefield, Ph.D., D.S.W.

Silver School of Social Work and Department of Psychiatry, New York University.

Regarding the Umpires: First, to avoid confusion, one has to distinguish the role of Umpire calls within the rules of baseball from the call as an attempt to state what happened. The Umpire calls them as he/she sees them, with the goal of getting it right - and understands that the way it looks can be misleading. But, whether correct or incorrect, the Umpire's call "stands" despite any later evidence that emerges to the contrary, and to that extent the call constitutes/constructs the game's reality. Diagnosis, too, has dual aspects - a game in which one plays by the rules to justify reimbursement, and a hypothesis about what is going on in the patient. I focus on the hypothesis-testing aspects of both Umpire calls and the DSM.

In attempting to make a call that reflects the truth, Umpires 1 and 3 embrace intellectual doctrines designed to deal with their epistemic anxieties - Umpire 1 can't stand uncertainty, and Umpire 3 can't stand the arrogance that comes from Umpire 1's certainty. Ironically, Umpire 1 and Umpire 3 fall into the same fallacy, that of collapsing ontology and epistemology into one. Umpire 1 naively sees his/her judgment as being a direct impression of reality without epistemic mediation, thus epistemological uncertainty is avoided. Umpire 3 sees his/her judgment as creating or constituting "reality" from his/her perspective, so again epistemological uncertainty is avoided. On the other hand, Umpire 2, while closest to the correct approach, describes his/her reality and his/her perception in a rather disconnected way.

So, I vote for Umpire 1.5 (humble realism): There are balls and there are strikes (plus some ontologically fuzzy cases), and based on how I see them and any other available evidence, I call them as I believe they are, and because the evidence in these cases is usually a pretty good indicator of reality, calling them as I see them usually equals calling them as they are. But, I can be wrong! The truth does not necessarily correspond to my call, and fresh evidence can always be brought to bear to help get closer to the truth.

Common sense offers the best guide here. Recently, Tigers' pitcher Armando Galarraga was one pitch away from achieving baseball immortality with a perfect game, an extremely rare event. In a close call at first base, Umpire Jim Joyce called the runner safe, destroying Galarraga's chance. But, as everyone saw from the instant reply, in fact the runner, Jason Donald, was out. Jim Joyce said to the press; "I just cost that kid a perfect game... I thought (Donald) beat the throw. I was convinced he beat the throw, until I saw the replay... It was the biggest call of my career and I kicked the (expletive) out of it." He then went to Galarraga and explained what he saw, and made it clear that he was wrong ("Imperfect" Umpire Apologizes by Steve Adubato, Ph.D., Star-Ledger). Fortunately for the lessons we and our kids take away from baseball, Joyce was not Umpire 1 or 2 or 3, but humble realist Umpire 1.5 who understood the possibility of error inherent in the attempt for mind to represent reality.

As to the other part of the question, the dichotomy between constructivism and realism is a false one. Our diagnostic categories are constructs (as are all concepts) intended in the long run to refer to underlying diseases/disorders. Current DSM diagnoses are constructs that are starting points for a recursive process aimed at getting at disorders. We somewhat misleadingly refer to them now as "disorders," although frequently we acknowledge that one of these categories likely encompasses many disorders. Close attention to the way revise our views and the grounds on which our judgments are made suggests that the individuation of disorders ultimately depends on the individuation of dysfunctions (see the answer to question 6).

### Commentary

Joseph Pierre, M.D.

UCLA Department of Psychiatry.

Consider the brief history of Pluto as a planet, as told in the recently published book, *How I Killed Pluto and Why It Had It Coming *[37]. A few thousand years ago, during the era of Greek geocentrism, the Earth was considered to be the center of the universe, while the sun and moon were regarded as two of the seven planets that orbited around it. Later in the 16^th ^century, as Copernicus' mathematical models of heliocentrism were embraced, the Earth and the sun traded categories at the expense of the moon. The subsequent discoveries of Uranus in 1781, Neptune in 1846, and Pluto in 1930 resulted in the total of nine planets that most of us learned about in elementary school. However, in 2006, Pluto was officially downgraded from classification as a planet, in part because of the discovery in 2005 of a larger mass of rock and ice called "Xena" orbiting not that far away. Now our children will be taught that there are only eight planets, and will perhaps eventually learn that there are also heavenly bodies called "dwarf planets," among them Pluto and Eris (the new, official name for "Xena").

To anyone that really relies on taxonomy in their daily work, it inevitably becomes apparent that such efforts at classification never seem to do a perfect job of "carving nature at its joints." This is especially true with scientifically-based taxonomies - they change based on the evolution of underlying definitions; new categories and sub-categories emerge while previous entities are re-categorized in order to accommodate new data; and challenges to classification at border-zones linger on. Although this kind of change sometimes causes the general public to regard science with skepticism, it is this very adaptability in the face of new data that is the strength of science and the feature that most distinguishes it from dogma.

The belief that this dynamic process is both acceptable and necessary for the Diagnostic and Statistical Manual of Mental Disorders (DSM) would seem to place myself in the category of Allen Frances' "Umpire #2," where I suspect the vast majority of clinicians reside. Still, since I have just suggested that reality often defies simple classification, allow me to state my position more clearly. I believe that psychiatric disorders do exist and that they are brain-mediated diseases (leaving aside for the moment the challenge of defining "disease") with genetic, biologic, and environmental etiologies and influences. The disorders (not diseases) cataloged in the DSM represent our best attempts at achieving consensus definitions of these conditions, seriously limited as we are by diagnosis that is based almost exclusively on describing manifest symptoms. Because of this limitation, it is unavoidable that psychiatric diagnosis is overly simplistic, just as many medical diagnoses would still be if not for technology-driven discoveries about pathophysiology. As such, DSM diagnoses are constructs, and DSM-IV's chief utility is as a "good enough rough guide for clinical work [38]."

As an imperfect work in progress, the DSM-IV contains diagnostic constructs of variable validity. In the tradition of Umpire #1, I believe that many of the disorders in DSM do a good job of describing the essential symptomatic features of what are probably "real diseases" (e.g. obsessive-compulsive disorder). However, I can also acknowledge the concerns of Umpire #3, including that some DSM disorders may tread dangerously close to pathological labeling of socially unacceptable behaviors (e.g. paraphilias) [39], while others might be better understood as "culture-bound syndromes" (e.g. anorexia) [40].

### Commentary

Gary Greenberg, Ph.D.

New London, CT.

"There are no balls or strikes until I call them" is not the postmodern fantasia that it sounds, nor is it a throwback to the idealism that Samuel Johnson refuted so thoroughly by kicking Bishop Berkeley in the knee. Or, to put it another way, it is neither the death knell of psychiatry nor a straw man for psychiatrists to use to refute their critics.

What it is, really, is just plain common sense. To question diagnosis is not to question the existence of suffering, or of the mind that gives us the experience of suffering, or of the value of sorting it into category. It is merely to point out that before we can do that sorting, we have to posit those categories. Where do they come from? Are there really diseases in nature?

Consider this question. What is the difference, from nature's point of view, between the snapping of a branch of an old oak tree and the snapping of a femur of an old man? We rightly recoil from the suggestion that there is no difference, and yet to assume that there is *in nature *a difference is to assume that nature cares about us enough to provide us with categories of broken hips. There is ample evidence, most stunningly Darwinian theory, that this is not true. Nature is indifferent. Unlike Major League Baseball, nature doesn't provide the rules by which the world can be divided into balls and strikes.

If there is a difference between the hip and the branch, it is surely to be found in the difference between the man and the tree, which is that the man is capable of caring about his femur, as are the people that love him. The only reason to distinguish one break from the other is to create a category--*intracapsular transcervical fracture, Stage II*, let's say. Naming the suffering, we bring it into the human realm. (It is not a coincidence that the authors of Genesis tell us that the first task given to Adam and Eve in Eden was to name the creatures of the earth; naming is how we put our stamp on the world.) By inventing categories like this one, we give ourselves a way to get hold of it, which in medicine means among other things a way to talk to other professionals about it, a way to determine treatment options, and a way to provide a prognosis to the patient and family. What we don't do is to discover that nature intends hips to break in certain ways, that there exist in nature intracapsular transcervical fractures and trochanteric fractures, any more than nature provides a branch with different ways to snap off a tree.

This much is uncontroversial, largely because whether you buy the argument or not, you are still going to treat the problem more or less the same way. The difference between fracture as a man made and a natural category is trivial, unless you're in a philosophical argument. But when it comes to psychiatry, something changes. To call a snapped femur an illness is to make only the broadest assumptions about human nature--that it is in our nature to walk and to be out of pain. To call fear *generalized anxiety disorder *or sadness accompanied by anhedonia, disturbances in sleep and appetite, and fatigue *depression *requires us to make much tighter, and more decisive, assumptions about who we are, about how we are supposed to feel, about what life is for. How much anxiety is a creature cognizant of its inevitable death supposed to feel? How sad should we be about the human condition? How do you know that?

To create these categories is to take a position on the most basic, and unanswerable, questions we face: what is the good life, and what makes it good? It's the epitome of hubris to claim that you have determined scientifically how to answer those questions, and yet to insist that you have found mental illnesses in nature is to do exactly that. But that's not to say that you can't determine scientifically patterns of psychic suffering as they are discerned by people who spend a lot of time observing and interacting with sufferers. The people who detect and name those patterns cannot help but organize what they observe according to their lived experience. The categories they invent then allow them to call those diseases into being. They don't make the categories up out of thin air, but neither do they find them under microscopes, or under rocks for that matter. That's what it means to say that the diseases don't exist until the doctors say they do. Which doesn't mean the diseases don't exist at all, just that they are human creations, and, at their best, fashioned out of love.

If psychiatry were to officially recognize this fundamental uncertainty, then it would become a much more honest profession--and, to my way of thinking, a more noble one. For it would not be able to lose sight of the basic mystery of who we are and how we are supposed to live.

### Commentary

Harold A Pincus, M.D.

Columbia University Department of Psychiatry.

The fourth umpire has a very pragmatic perspective and understands that a classification of diagnostic categories is used for many different purposes by many different groups and individuals. Umpire 4 also understands that these various "user groups" approach their tasks with varying empirical, philosophical and historical backgrounds and, and with this proliferation of users and backgrounds, there needs to be a balance between (to mix metaphors) letting "a thousand flowers bloom" - creating a Tower of Babel with little ability to effectively communicate among these groups - and a single approach that cannot be tailored to particular needs. From this perspective, there is a recognition that the world has changed and the management of information has become the pre-eminent task of a classification system, overshadowing (but also enhancing), the clinical, research and educational goals of a classification. As such, the ICD/DSM should serve a critical translation function to anchor communications among multiple user groups that apply psychiatric classification in their day to day functions.

This information management goal intersects with multiple user groups in terms of:

-health policy

-clinical decision making

-quality measurement

-epidemiology

-educational certification/accreditation

-multiple areas of research from genetics to psychopharmacology to cognitive science, etc.

The way this would work is that the ICD/DSM classification would remain relatively stable, serving as a kind of "Rosetta Stone" to facilitate communication among the various user groups. Each individual user "tribe" (or individual scientist) would be free to identify various alternative classifications. However, all journals or other public reporting mechanisms would require that any clinical population also be described in the ICD/DSM classification in addition to whatever tribal criteria for the "Syndrome XYZ", 70% met ICD/DSM criteria for GAD, 40% OCD, and 30% Anxiety Disorder, NOS). Changes in future (descriptive) classifications should be infrequent and guided by a highly conservative process that would only incorporate changes with strong evidence that they:

1. Enhance overall communication among the "tribes"

2. Enhance clinical decision-making

3. Enhance patient outcomes

However, ICD/DSM would have a section describing the relationships among the various tribal concepts that could be updated on a more frequent basis.

Note that this approach gives up the ideal (or even a focus) on validity, per se. Maintaining effective communication (most notably, effective use, reliability and understandability) and clinical utility [41] (either the more limited improvement of clinical and organizational decision-making processes or the ideal of outcomes improvement) become the principal goals of the classification. In other words, while a psychiatric classification must be useful for a variety of purposes, it cannot be expected to be simultaneously at the forefront of, for example, neurobiology and genetics, psychoanalysis, and the education of mental health counselors, primary care providers and psychologists.

However, multiple groups can continue their work on epistemic iteration using genetic approaches and others can develop ways to better measure quality or costs of care and yet others can study dimensional ratings of personality. However, each tribal group would need to be able to communicate across the commons using the "Rosetta Stone". Thus, we would not be wobbling toward the asymptote of true validity, but, instead, be very slowly, but continually, rising toward the goal of better outcomes for patients.

### Commentary

Thomas Szasz, M.D.

SUNY Upstate Medical University.

I thank Dr. James Phillips for inviting me to comment on this debate. I am pleased but hesitant to accept, lest by engaging in a discussion of the DSM (the American Psychiatric Association's *Diagnostic and Statistical Manual of Mental Disorders*) I legitimize the conceptual validity of "mental disorders" as medical diseases, and of psychiatry as a medical specialty.

Psychiatrists and others who engage in this and similar discussions accept psychiatry as a science and medical discipline, the American Psychiatric Association (APA) as a medical-scientific organization, and the DSM as a list of "disorders," a weasel word for "diagnoses" and "diseases," which are different phenomena, not merely different words for the same phenomenon.

In law, the APA is a legitimating organization and the DSM a legitimating document. In practice, it is the APA and the DSM that provide medical, legal and ethical justification for physicians to diagnose and treat, judges to incarcerate and excuse, insurance companies to pay, and a myriad other social exchanges to be transacted. Implicitly, if not explicitly, the debaters's task is to improve the "accuracy" of the DSM as a "diagnostic instrument" and increase its power as a document of legitimation.

Long ago, having become convinced of the fictitious character of mental disorders, the immorality of psychiatric coercions and excuses, and the frequent injuriousness of psychiatric treatments, I set myself a very different task: namely, to delegitimize the legitimating authorities and agencies and their vast powers, enforced by psychiatrists and other mental health professionals, mental health laws, mental health courts, and mental health sentences.

In *Psychiatry: The Science of Lies*, I cite the warning of John Selden, the celebrated seventeenth-century English jurist and scholar: "The reason of a thing is not to be inquired after, till you are sure the thing itself be so. We commonly are at, *what's the reason for it? *before we are sure of the thing." In psychiatry it is usually impossible to be sure of "'what a thing itself really is," because "the thing itself" is prejudged by social convention couched in ordinary language and then translated into pseudo-medical jargon.

Seventy-five years ago, in my teens, I suspected that mental illness was a bogus entity and kept my mouth shut. Twenty-five years later, more secure in my identity, I said so in print. Fifty years later, in the tenth decade of my life, I am pleased to read Dr. Allen Frances candidly acknowledging: "Alas, I have read dozens of definitions of mental disorder (and helped to write one) and I can't say that any have the slightest value whatever. Historically, conditions have become mental disorders by accretion and practical necessity, not because they met some independent set of operationalized definitional criteria. Indeed, the concept of mental disorder is so amorphous, protean, and heterogeneous that it inherently defies definition. This is a hole at the center of psychiatric classification." This is as good as saying, "Mental illness, there ain't no such thing," and still remain loyal to one's profession.

The fallacy intrinsic to the concept of mental illness - call it mistake, mendacity, metaphor, myth, oxymoron, or what you will - constitutes a vastly larger "problem" than the phrase "a hole at the center of psychiatric classification" suggests. The "hole" - "mental illness" as medical problem - affects medicine, law, education, economics, politics, psychiatry, the mental health professions, everyday language - indeed the very fabric of contemporary Western, especially American, society. The concept of "psychiatric diagnosis," enshrined in the DSM and treated by the discussants as a "problem," is challenging because it is also a solution, albeit a false one.

Medicalization, epitomized by psychiatry, is the foundation stone of our modern, secular-statist ideology, manifested by the Therapeutic State. The DSM, though patently absurd, has become an utterly indispensable legal-social tool.

Ideologies - supported by common consent, church, state, and tradition - are social facts/"truths." As such, they are virtually impervious to criticism and possess very long lives. The DSM is here to stay and so is the intellectual and moral morass in which psychiatry has entwined itself and the modern mind.

### Commentary: On Inviting the Gorilla to the Epistemological Party

Elliott Martin, M.D.

Yale University Department of Psychiatry.

What makes the epistemological umpire analogy so enticing is its capacity for adaptation, the fact that the strike zone must be different for every batter. If I call 'em as I see 'em, then of course what is a ball thrown to one batter may be a strike thrown to another. As applied to the broadly descriptive nosology of DSM IV there is hardly an argument to be made against this. But let's add a missing piece to the scenario. Let's cast the eight hundred pound gorilla in the analogy, the insurers, as 'the owner'. More specifically, let's call the beast 'the hometown owner'. And then let's say the umpire's salary is paid by the owner.

With the game yet played on rural fields, before the advent of electronic pitch-tracking devices, before the price of every pitch was calculated, before the global media contracts, the strike zone was a sacred space, the tiny, arbitrary, marked off piece of ether from which intimacy the entire game was decided. Before the 'owners' blew the entire field up to stadium-size the game was about conceptualization and process; before psychiatry was snatched up by the insurers the pathologies were sought in subjectivity over objectivity. Artfulness existed alongside science. What, after all, did psychiatrists care for nosology before the rise of private insurance over the past several decades? Disordered thinking, as opposed to ordered thinking, was just that. Slapping a name on it did little to change the fact. One man's depression is another man's 'blues', and what does the patient care for the label?

'Carving nature' does require a measure of reliability, true, but the only conversations I have had in which I have coughed up the full DSM criteria have been those over the telephone, most often in the emergency room, with insurance reviewers 'objectively' determining, from up to thousands of miles away, whether a particular patient warrants two days or three days in which to be cured. And at that, for the benefit and safety of my patients, my strike zone widens tremendously after five minutes, and my diagnoses tend to reduce to the very non-DSM, if at times heavily punctuated, 'imminently suicidal!' or 'imminently homicidal!'. The arguments tend to end there, and it is apparent that what is missing in the epistemological umpire analogy is the hard baseball rule against arguing balls and strikes.

As a former academic, however, I simply have to believe that there is an inherent value in the pursuit of knowledge for knowledge's sake, that all sciences, veiled or not, are interwoven, regardless of the current paradigms, and the loss of even one is somehow crippling to the others. But 'the owners', despite the fact that they stand oblivious, willfully or not, to the devastation they create, can no longer be ignored in these arguments. Whatever the historical mechanisms, the pursuit of knowledge has come up hard against the pursuit of profit in these last few decades. I contend that the process of classification is the process that, if not created by, than at least has been manipulated ever since by the owners. As students of the human mind, arbitrary classification of disorders of the mind does not inform us; it informs the gorilla. Describing 'normalcy' and 'variants thereof' only serve to destroy further an already hobbled subjectivity. Nosology destroys narrative, and where formerly our patients were more appropriately likened to novels, they are now become, for the ease of illiterate overlords, more like newspapers.

As the noted Assyriologist, Jean Bottero, put it in defense of his own limited field, "Yes, the university of sciences is useless; for profit, yes, philosophy is useless, anthropology is useless, archaeology, philology, and history are useless, oriental studies and Assyriology are useless, entirely useless. That is why we hold them in such high esteem!" [[42], p. 25] Psychiatry finds itself in a unique position among the 'useless' sciences. Like the umpire offered a bribe by the owner, if the field chooses utterly to subserve profit it likely stands to gain tremendously. If the field chooses to uphold an ideal of humanism in the face of gorilla-ism then we will likely be faced with the same fate as philosophy, anthropology, archaeology, philology, and history. In which case let us all call 'em as we see 'em, keep the paperwork tidy, and at the very least be ever mindful of the watchful gaze of the gorilla.

### Allen Frances responds: There Is A Time And Place For Every Umpire

None of the five umpires is completely right all of the time. And none is totally wrong all of the time. Each has a season and appropriate time at the plate.

Forty years ago, Umpires 1, 3, and 5 were in competitive ascendance. The nascent school of biological psychiatry was a confident Umpire 1- convinced that mental disorders would soon yield their secrets and be as fully understood as physical illnesses. In fact, there was a heated controversy whether the new diagnostic manual (DSM III) then being prepared was a catalog of 'disorders' or of their much preferred term 'diseases'.

In sharp contrast, the competing models that dominated psychiatry forty years ago were very much like the skeptical Umpires 3 and 5- in their different ways, all were nihilistic about the value or reality of psychiatric diagnosis. Psychoanalysis dealt with highly inferential concepts impossible to reduce to reliable diagnosis. Family, group, and community psychiatry went so far as to deny that the individual patient was a proper or very relevant unit for diagnostic assessment, preferring models that diagnosed the system at larger aggregates of interpersonal affiliation. When Szasz, then as now, decried the 'myth of mental illness', there was little coherent opposition outside the group of the smugly confident pioneers of biological psychiatry (who soon would be hoisted by their own petard).

The years have not been kind to umpires 1, 3, and 5. Each still stakes some small claim to attention, but umpire 2 now clearly rules and welcomes the collaboration of his close cousin, the ever practical umpire 4.

Why the revolution in epistemological sentiment? Biological psychiatry helped spark a wondrous neuroscience revolution that is perhaps the most thrilling focus of twenty first century biological science. But the findings have revealed a remarkably complex brain unwilling to yield any simple answers. There is thus far almost no translation from the glory of basic science discovery to the hard slog of understanding the etiology and pathogenesis of the 'mental disorders'. These no longer seem at all reducible to simple diseases, but rather are better understood as no more than currently convenient constructs or heuristics that allow us to communicate with one another as we conduct our clinical, research, educational, forensic, and administrative work.

Most hard core biological psychiatrists have lost heart in the naςve faith of umpire 1 that he can define simple models of illness. Those who were hunting (and reporting) the gene or genes for schizophrenia, bipolar, and other disorders have been forced repeatedly to retract and eat humble pie. Initial findings never achieved replication for what became the obvious reason that there is no 'disease' of schizophrenia- that instead schizophrenia is better understood as just a construct (albeit it a very useful one) with hundreds of different 'causes'.

Meanwhile the diagnostic nihilism of Umpires 3 and 5 also became less relevant when DSM III proved that psychiatric diagnosis could be a reliable and useful tool of communication.

Umpire 2 now rules. Mental disorders are no more and no less than constructs. And Umpire 4 is quick to point out that they are very useful constructs. The current dominance of Umpires 2 and 4 is temporary, and certainly not complete. In some very gradual and piecemeal way, the future holds hope for an increased role for Umpire 1. As we slowly discover the biology of mental disorders, small subunits will cohere around a common pathogenesis and declare themselves as a disease. This is beginning to happen for the dementias of the Alzheimer's type. But it will always be necessary to retain the corrective voices of the skeptical Umpires 3 and 5- to remind us just how little we know and how feeble are our tools for knowing.

#### Reply to Drs Zachar and Lobello

Thank you for your contribution which I received after writing my own. You have stated my position with much greater clarity and erudition than I could muster.

#### Reply to Dr Pouncey

Thank you for your clarification of the Umpire metaphor. Your analysis nicely demonstrates the similarities and the differences in the positions of Umpires 1 and 2- both accept the possibility of an independent reality, but differ sharply in there estimation of our current ability to apprehend it.

#### Reply to Dr Ghaemi

Dr Ghaemi sets up a false and totally unnecessary dichotomy between his true believer version of realism and what he calls "taking a random walk". It is possible, indeed necessary, to take a very modest position regarding the current state of certitude of psychiatric knowledge on the causes of psychopathology without assuming that we know nothing or are walking totally blind or that our constructs have no current heuristic value. Umpire 2's honest admission that he can do no better than call them as he sees them does not deny the possibility of real strikes and real balls- it just states the very constrained limits of our apprehension. I have no problem at all with the metaphor of epistemic iteration- it is obviously the route of all science. But let's realize how early in the path we are and how uncertain is its best direction.

#### Reply to Dr Cerullo

How comforting to be a first umpire. I admire the magisterial confidence of Dr Cerullo's statement, "Most working scientists and philosophers would be classified as modern realists who believe there is an independent objective external reality". I wish I could feel so firmly planted in a "real" world and possess such naςve faith in mankind's capacity to apprehend its contours. Alas, as I read it, the enormous expansion of human knowledge during the last hundred years is enough to make umpire 1's head spin with confusion. The more we learn, the more we discover just how much we don't (and perhaps can't) know. Einstein gave us a four dimensional world that even physicists have trouble visualizing. Then the string theorists made it exponentially more complicated by expanding the dimensions into double figures and introducing conceptions of reality that may or may not ever be testable. The quantum theorists describe a "spooky" (Einstein's term) and inherently uncertain world that lends itself to extremely accurate large n prediction, but totally defies our intuitive understanding of the specific mechanics. It also turns out that we are pathetically limited in our sensory capacities, even when they are extended with our most powerful sensing instruments. Evolution allows us to detect only 4% of our universe, the rest of energy and matter being "dark" to us. Indeed, there may be a vast multiplicity of multiverses out there and we may never know them. So I don't see human beings as having great status as judges of reality- we are like mice describing the proverbial elephant- having available only fallible and very temporary constructs.

To get back to our umpires, the connections between brain functioning and psychiatric problems are definitely real, but they are so complex and heterogeneous as to defy any simple "realist" faith that we are close to seeing them straight on, much less solving them.

#### Response to Dr Wakefield

Drs Wakefield and Pouncey have made many of the same important points. Dr Wakefield's "humble realism" (associated with an honest and flexible willingness to admit fallibility and the possibility of error) works for a great baseball umpire and is not a bad model for a psychiatric diagnostician. The difference between umpire 2 and umpire 1.5 depends on how close you think our field is to understanding the reality of psychopathology. I am even more humble than Dr Wakefield and will stick with umpire 2.

#### Reply to Dr Pierre

I agree.

#### Reply to Dr Greenberg

In defending Umpire 3, Dr Greenberg assumes a grandly, neutral view of man's place in the world and makes clear how limited are our abilities in naming and classifying its manifestations. Greenberg rightly suggests that the distinction between a broken branch and a broken femur may be extremely meaningful to the patient and his doctor, but is really trivial in the grand scheme of an indifferent nature. He might equally have pointed out that from a bacteria's perspective, pneumonia is not a disease- it is just an opportunity for a good feed. Diseases, according to Greenberg's argument, are no more than human constructs made up de novo by us as inherently self interested third umpires.

From Greenberg's lofty perch, mankind's attempts to label do seem pathetically self referential and solipsistic, extremely limited in their apprehension of reality (even assuming that there is a graspable reality ready to be apprehended). But it seems to me that his level of philosophic detachment works only in the exalted theoretical realms, and contrary (to his statement) fails badly to do justice to the needs and opportunities of our everyday, "common sense" world.

Greenberg and I do agree completely on several points: 1) if mother nature had the gift of speaking our language and the motivation to do so, she would probably indicate she couldn't care less about our names and that she doesn't feel particularly well described by them; 2) our categories are no more than tentative approximations and are subject to distortion by personal whims, cultural values local to time and place, ignorance, and the profit motive; and, 3) psychiatry's names should be used with special caution because they lack strong external validators, carry great social valence, and describe very fuzzy territorial boundaries.

Where my umpire 2 position differs from Greenberg's umpire 3 is in our relative estimations of how closely our names and constructs can ever come to approximating an underlying reality. My umpire 2 position is skeptical about umpire 1's current ability "to call them as they are" and advises modesty in the face of the brain's seemingly inexhaustible complexity. But I remain hopeful that there is a reality and that, at least at the human level, it will eventually become more or less knowable. We may never fully figure out the origin and fate of the universe or the loopy weirdness of the quantum world. But the odds are that decades (or centuries) of scientific advance will gradually elucidate the hundreds (or thousands) of different pathways responsible for what we now crudely call "schizophrenia".

Greenberg is more skeptical than I about the progress of science and is, at heart, a platonic idealist who finds life cheapened by excessive brain materialism. He sees psychiatric disorders as no more than human constructs - metaphors, some of which are useful, some harmful. His umpire 3 does not does not believe the glory and pain of human existence can or should be completely reduced to the level of chemical reactions or neuronal misconnections. This is a fair view for poets and philosophers (and Greenberg is both), but I see a ghost in his machine and dispute that allowing it in makes "common sense".

#### Reply to Dr Pincus

Thank you for inventing the fourth umpire. Dr Pincus is the most practical of men and he has created a handy metaphor for describing the ultimate goal of any DSM- to be useful to its users. There is only one problem with the fourth umpire's position- but it is a big one. There is no external check on his discretion, no scientific or value system that guides what is useful. Everything depends on the skill and goodwill of the umpire. In the wrong hands pragmatism can have dreadful consequences- commissars who treat political dissent as mental illness or judges who psychiatrically commit run of the mill rapists to keep them off the streets. But to ignore the practical consequences of psychiatric decisions leads to its own set of abuses- most recently diagnostic inflation and excessive treatment.

#### Reply to Dr Szasz

I have enormous respect for intellectual reach and depth of Dr Szasz' critique of psychiatric diagnosis and for the moral power of his lifelong efforts to prevent its misuse. He skillfully undercut the pretentions of the Umpire I position at a time when its biological proponents were at their triumphalist peak, loudly trumpeting that they were close to finding the gene for schizophrenia and to elucidating its brain lesions. He anticipated and exposed the naivete of these overly ambitious and misleading claims. He has fought the good fight to protect the rights, dignity, and personal responsibility of those deemed to be "mentally ill". My argument with Dr Szasz is that he goes too far and draws bright lines where there are shades of gray. Surely, he is right that schizophrenia is no "disease", but that does not mean it is a "myth". Surely, he is right that psychiatric diagnosis can be misused and misunderstood, but that doesn't mean it is useless or can be dispensed with. Dr Szasz is correct in defining the many of problems with psychiatric diagnosis, but doesn't have alternative solutions. There is a baby in there with the bath water he is so eager to discard.

#### Reply to Dr Martin

I agree that we can't always assume the Umpires are acting only from the purist and most disinterested of motives. Games can be fixed for financial gain and psychiatry operates in a real world of large drug company, insurance, and publishing profits. My experience has been that the actual framers of DSM IV and of DSM 5 have not been shills for industry- but that heavy drug marketing has led to much over-diagnosis using DSM IV and that the risks are greatly heightened because of the new diagnoses being suggested for DSM 5. Dr Martin's comment makes clear that we must be aware the diagnosis of a given patient can be distorted by real world economic factors and must be ever vigilant to protect the integrity of the process.

## Question #2: What is a Mental Disorder?

*It has been difficult to reach agreement on a definition of mental disorder. Could you comment on this problem, or offer what you think is an adequate definition of the concept, mental disorder?*

### Introduction

On the face of it this is a strange question. As treating clinicians we surely should be able to offer a definition of what it is we treat. As researchers surely we should be able to define the object of our research. And finally as philosophers writing about mental illness, surely we should be able to provide a definition of the object of our investigation. So why is it so difficult to accomplish these tasks? Allen Frances has puzzled over this question, and as he indicates below, it leads him into Humpty Dumpty's world of "shifting, ambiguous, and idiosyncratic word usages."

Failures to accomplish a consensually accepted definition lead in two directions: give up or keep trying. The first approach is represented by Warren Kinghorn, who argues in his commentary that we won't achieve the desired definition and don't need it anyway - any more than other specialists do for their work - and thus should abandon the effort to try yet again to get it right in DSM-5.

The opposite approach is represented in different ways: by Jerome Wakefield, on the one hand, and Stein and colleagues, on the other. In his contribution to this article Wakefield presents the evolution-based harmful dysfunction definition of mental illness for which he is justly well-known. In this contribution he argues that the varied positions of figures like Allen Frances and Kenneth Kendler depend implicitly on the HD understanding and definition of mental disorder.

Stein and colleagues (not represented in this article) [43] take another approach in trying to improve the DSM-IV definition by operationalizing it, and then going to work on the operationalized definition. They tweak some of the DSM-IV (definitional) criteria as well as adding further criteria, e.g., acknowledging the normative, value-laden aspect of many diagnoses. In their effort to improve the DSM-IV definition, they address many of the complaints lodged against DSM-IV (co-morbidity, poor separation between diagnoses, poor separation from normality, etc.) In trying to be comprehensive, they include issues of clinical utility, scientific accuracy through validators, and pragmatic concern for patient outcome. They do not, however, deal with the issue discussed in Question 5, namely, that these can be conflicting agendas, and that at times we effectively prioritize one over the other.

Life never being as neat as one would like it, Allen Frances and Joseph Pierre defeat my effort of a simple dichotomy and occupy a middle ground between the alternatives of giving up on a definition or trying to improve it. Each argues for attempting a definition but assures us that it will be a messy undertaking.

And that conclusion reminds me that in this search for an adequate definition of mental disorder it would be useful to invoke Wittgenstein's notion of family resemblances. In discussing the essence of language or language games, Wittgenstein writes:

Instead of producing something common to all that we call language, I am saying that these phenomena have no one thing in common which makes us use the same word for all,-but that they are *related *to one another in many ways. And it is because of this relationship, or these relationships, that we call them all "language."...

I can think of no better expression to characterize these similarities than "family resemblances"; for the various resemblances between members of a family: build, features, colour of eyes, gait, temperament, etc. etc. overlap and criss-cross in the same way.-And I shall say: 'games' form a family. [[44], pp. 31-32].

The Wittgensteinian approach would represent the middle ground of Frances and Pierre. In technical DSM terminology, diagnoses don't all share the same properties or validators; they resemble one another because they share some. A diagnosis might have a place in the nosology because of "historical accretion," but might lose it because it doesn't meet standards of other current validators. Examples are the paraphilias and conduct disturbances, both of which may lose their admission tickets because of excessive normative valence and inadequate internal distress. Borderline Personality Disorder (now Borderline Type) is being retained presumably because descriptively it covers a lot of the symptomatic morass otherwise not covered; but with its known problems - heterogeneous presentation, excessive comorbidity, lack of genetic or pathophysiologic foundation - it will probably collapse and be carved up eventually for lack of real validators, i.e., not enough validators in common with other members of the DSM family. And finally, to underline Pierre's point, distinguishing a validator from a value is an ambiguous enterprise.

I readily acknowledge that the family resemblance model will not satisfy those in search of a tighter definition of what constitutes a psychiatric disorder. Its main virtue is that it reflects how we actually "define" - and in the absence of a tighter definition, will continue to "define" - psychiatric disorders.

### Commentary

Jerome C. Wakefield, Ph.D., D.S.W.

Silver School of Social Work and Department of Psychiatry, New York University.

Al Frances's powerful writings on psychiatric diagnosis offer vigorous arguments about what should and should not be diagnosed as psychiatrically disordered (not only regarding DSM-5 overreaching; consider his adding the clinical significance criterion to most diagnostic criteria sets in DSM-IV). Yet, Frances vehemently denies that there is any coherent concept underlying our judgments of what is and is not a disorder. This may save him from a troublesome additional debate, but, as observed by some commentators, it undercuts the coherence let alone force of his critique of false-positive implications of DSM-5 proposals.

Despite his disavowals, Frances's arguments derive their enormous power from an implicit reliance on common intuitions about the concept of disorder as failure of biologically designed human nature. Sometimes this implicit appeal emerges explicitly, as in Frances's [45] explanation of why he rejects DSM-5's proposed approach to behavioral addiction: "The fundamental problem is that repetitive (even if costly) pleasure seeking is a ubiquitous part of human nature.... The evolution of our brains was strongly influenced by the fact that, until recently, most people did not get to live very long. Our hard brain wiring was built for short term survival and propagating DNA- not for the longer term planning that would be desirable now that we have much lengthened lifespans.... This type of hard wiring was clearly a winner in the evolutionary struggle when life was "nasty, brutish, and short". But it gets us into constant trouble in a world where pleasure temptations are everywhere and their long term negative consequences should count for more than our brains are wired to appreciate."

Notice that Ken Kendler et al. [46], on the extreme opposite side of the DSM-5 debate, implicitly appeal to the same biological-design criterion when explaining why fearful distress in reaction to real danger is not a disorder: "An individual experiencing a panic attack after just barely escaping a fatal climbing accident would not be considered psychiatrically disordered because the mechanism for panic attacks probably evolved to prepare us for such situations of real danger" (p. 771).

So, what is the concept of disorder to which Frances and Kendler implicitly appeal? The DSM's definition of disorder says that a disorder exists only when symptoms are caused by a dysfunction in the individual and lead to certain forms of harm, such as distress or impairment. Observing that the concept of "dysfunction" was left unelaborated and that distress or disability are not the only harms that would warrant diagnosis, I proposed what I labeled the "harmful dysfunction" (HD) analysis of the concept of disorder [47-52].

The harmful dysfunction analysis maintains that the concept of disorder has two components, a factual component and a value component. To be a disorder, a condition must satisfy both components. The value or "harm" component refers to negative or undesirable or harmful conditions, which applies to most symptomatic conditions. Obviously, who gets to make the judgment that a condition is harmful and on what grounds (especially in a pluralistic society) is a complex issue. But the basic point is that no condition, even if a clear biological malfunction, is a disorder if it is not considered in some sense harmful to the individual or society. This is the basis for the "clinical significance" requirement.

The factual component requires that the condition must involve a failure of some mental mechanism to perform one of its natural, biologically designed functions. This is highly inferential and speculative and fuzzy at this stage of knowledge of mental processes, but it is the conceptual target at which we aim nonetheless. Indeed, although both the notions of dysfunction and harm are fuzzy concepts, as long as they determine a range of clear cases on either side of the disorder/non-disorder boundary, they can provide a cogent and useful conceptual structure. Other useful categorical distinctions - such as between night and day, or child and adult - also have fuzzy boundaries, and pragmatic considerations determine specifically where the dividing line is drawn (see Question 1).

Today, we understand that human nature -specifically, species-typical biological design -- is due to evolution through natural selection. So, dysfunction in the sense relevant to judgments of medical disorder consists of failure of internal mechanisms to perform evolved functions. The "dysfunction" component of the analysis means that, as far as the legitimate application of the concept of disorder goes, disorder cannot be manufactured from personal or social values and used as a cover for "treatment" in service of social control. The "dysfunction" requirement places a limit on what can be legitimately said to be a disorder, and explains why many negative conditions are false positives.

How can we test this account of the generally shared meaning of our distinction between disorder and non-disorder? One way is to see if it is consistent with some of our shared intuitions. Consider some simple examples of conditions NOT considered disorders. Neither illiteracy nor an immigrant's inability to speak the local language are considered disorders, yet are terribly impairing, potentially distressing, and disadvantageous mental conditions (whereas dyslexia and aphasia are disorders). Being a "night person" rather than a "morning person" in a 9-to-5-structured culture is potentially disadvantageous, but considered a normal variation. Fertility when pregnancy is unwanted, pregnancy when children are unwanted, and pain during childbirth are all undesirable and potentially harmful conditions commonly treated by physicians but not considered disorders. Neither debilitating fatigue after exertion nor sleep - probably the single most massively impairing human condition of all, rendering virtually everyone semi-paralyzed and periodically hallucinating for one-third of their lifespans -- are seen as disorders. Nor is delinquent behavior by rambunctious teenagers or grief after a loved one's death.

The source of these classificatory judgments cannot be reduced to personal or social values. Many of these non-disordered conditions are personally and/or socially undesirable. There is some additional element operating here to explain these judgments. The common element is that we consider all these conditions to be part of the way human beings are designed to function, however problematic they may be in our current environment.

Moreover, our intuitions are that entire cultures can be incorrect about their disorder judgments. Victorians' deeply held values and beliefs led them to classify masturbation and female clitoral orgasm as disorders, some ante-bellum Southern physicians considered slaves who ran away from their masters to be psychiatrically disordered ("drapetomania"), and Soviet psychiatrists treated political dissidents as disordered. We believe that these diagnostic judgments, although consistent with the respective cultures' values, were incorrect -- not correct "for them" and incorrect for us, but just plain wrong. The factual component of "disorder" explains how this can be so.

Now, consider the types of conditions to be found in the DSM. At this point in the history of psychiatry, we are about where Hippocrates was in formulating diagnostic categories. We know virtually nothing about the underlying natures of mental mechanisms, but from circumstantial evidence and indirect inferences, we infer what conditions are likely disorders. The superordinate categories in the DSM - e.g., psychotic (thought) disorders, anxiety disorders, mood (sadness/elation emotion) disorders, sexual disorders, sleep disorders, and so on - correspond to human systems about which we feel we can pretty reliably infer that they are biologically designed to operate in certain ways, and we can recognize in a range of cases when something has gone wrong - albeit with large fuzzy boundaries and large ranges of uncertain cases given our ignorance.

Another way of assessing the HD analysis is by whether it accomplishes certain important goals motivating such an analysis. At a minimum, an analysis of the concept of mental disorder should do four things:

(1) Explain widely shared classificatory judgments about whether or not problematic conditions are disorders.

(2) Explain why mental disorders are disorders in the same sense as physical disorders, thus why psychiatry is part of medicine.

(3) Explain the distinction between control of socially undesirable mental conditions versus treatment of mental disorder.

(4) Offer a fruitful way of thinking about research.

The first three goals are addressed above. As to research, the HD analysis explains the primary goal as seeking to understand mental mechanisms and their functions, and ultimately to identify and distinguish specific mental dysfunctions, yielding an "etiological" classification.

Frances's end run around the concept of disorder is accomplished partly by his insisting on a scientific-sounding "cost-benefit analysis" of each DSM-5 proposal. This is a useful rubric for raising concerns and is of course an improvement over wanton unreflective pathologization. In drawing boundaries in some fuzzy domains, the concept of disorder offers little guidance, and perhaps cost-benefit analysis is all we can do. However, generally reframing the process of deciding whether to pathologize a condition as a cost-benefit analysis, if taken seriously, is not only intellectually unsupportable but dangerous, for the same reasons why one would not want cost-benefit analysis to determine whether an accused was judged guilty in a trial - namely, one loses any protection against social control aspirations. After all, for all we know a careful cost-benefit analysis might show that from a social perspective "treating" the Soviet dissidents for psychosis, slaves who ran away for "drapetomania," and women for "pathological" clitoral orgasms was justifiable in the respective social circumstances--but it was incorrect use of diagnosis nonetheless, because the individuals so labeled (in many cases) did not actually suffer from disorders. Just as the health professions spring from the concept of disorder, restraint of efforts to use the health professions for social control also spring from this crucial concept. To deny the existence and importance of the concept of mental disorder in a discussion of the validity of diagnostic criteria in psychiatry is sort of like a teacher denying there is knowledge or ignorance before entering the classroom or a judge denying there is criminal guilt or innocence before rendering a verdict.

I believe that, despite his misstep on the concept of disorder, Frances is entirely correct in his relentless focus on false positives as the Achilles heel of the DSM-5 effort. The reason why this is so is not simply a matter of preventing repetitions of the "false epidemics" of the past due to criteria revisions. The deeper point is that the very aspirations of the editors of DSM-5 for a paradigm shift towards etiologically based diagnosis - a shift for which we are not yet ready - depends in the long run on distinguishing etiologies. That means distinguishing different dysfunctions as well as distinguishing normal non-dysfunctions from dysfunctions that underlie similar symptom presentations.

Because we are not yet ready to distinguish dysfunctions, DSM-5 must remain theory neutral. However, a serious conceptual-validity review could have allowed us to do considerably better in distinguishing dysfunction from likely non-dysfunction for many categories. Instead, the DSM-5 work groups are expanding diagnoses so that more individuals coming into consultation can be labeled with a disorder, without careful conceptual analysis. Given this focus, the DSM-5 is likely to take psychiatry further away from the editors' stated goal. The DSM-5 could have made progress towards etiologically grounded categories by eliminating some of the false positives that afflict the manual's operational criteria sets, placing them under separate V Code categories. With false positives swamping many categories, psychiatric science will continue to flounder, unable to distinguish dysfunction etiologies because its criteria cannot in many cases even distinguish internal dysfunction where something has gone wrong from intense but normal biologically designed reactions to events.

### Commentary: Definitions of "Mental Disorder:" Elusion and Illusion

Warren Kinghorn, M.D.

Duke University Department of Psychiatry.

The definitions of "mental disorder" which have appeared in each edition of the *DSM *since *DSM-III *(hereafter referred to as the "*DSM *definitions") are both carefully crafted and widely ignored. Pioneered by Spitzer and Endicott as a means for demarcating (true) mental disorders from non-pathological conditions in the wake of controversy over the status of homosexuality in *DSM-II *[53], the *DSM *definitions have from the beginning, and despite copious qualification and caveat, proved controversial and largely irrelevant to the practical use of the *DSM*. Specific criticisms of the *DSM *definitions, such as their circular use of "clinically significant," are by now commonplace [54,55]. The *DSM-5 *Task Force has proposed an updated definition which seeks to improve on prior definitions and which emphasizes the pragmatic and method-driven nature of psychiatric nosology [43]. The *DSM-5 *proposal is worthy of sustained critical consideration which I do not offer here. In this brief account, rather, I wish to offer three reasons why future editions of the *DSM *should not include a definition of "mental disorder." In each case, I argue, the *DSM *definitions *seem *to contribute to a certain good, but this is illusion.

First, the *DSM *definitions *seem *only to describe the terrain of what is already considered "mental disorder," innocuously supplying rough logical boundaries to psychiatric praxis without limiting or shaping it. But this is an illusion. Although, as Sadler [54] points out, the *DSM *definitions have not in practice influenced the way that new diagnoses are incorporated into the *DSM*, they do provide a regulative language for how one speaks about the mental disorders that are already there. Questions, for example, about whether major depressive disorder most properly inheres in an individual or in a group (or even in a society; [56]) run counter to the methodological individualism of the *DSM*, enshrined in its definitions of mental disorder, and are therefore difficult to ask without bringing the entire *DSM *project into question. This should not be the case; it is precisely by excluding useful questions that the *DSM *renders itself an obstacle to nosological advance rather than a catalyst to it.

Second, the *DSM *definitions of mental disorder *seem *to demarcate a safe conceptual territory within human life and experience within which the medical model can properly rule. The unspoken but nonetheless persuasive model seems to run something like: *Why should a psychiatric technology (medication, ECT, manual-driven psychotherapy, etc.) be deployed within this situation? *A: Because it's a mental disorder, and one uses psychiatric treatments for mental disorders. *Q: But how do we know that this is a mental disorder? *A: Well, it's in the *DSM*, and besides, it fits into the conceptual space which the *DSM *defines as "mental disorder." But this, too, is illusory and deceptive. The deployment of psychiatric technology is not justified because a particular condition is demarcated as "mental disorder" but because, after all goods are weighed and all options considered, the use of technology is prudentially indicated. Whether the condition is classified as "mental disorder" has little to do with this particular question. Although the *DSM *definitions importantly exclude certain situations (e.g., primary social deviance) from the medical model, it would be better for the *DSM *simply to stipulate these ethical commitments rather than to embed them within the definition of "mental disorder." Moreover, the fact that a certain condition satisfies the *DSM *definitions does not serve as *prima facie *justification of the deployment of technology for that condition, and the *DSM *should not collude in any contrary assumption.

Finally, the *DSM *definitions *seem *to focus diverse mental health professionals on a common moral project. Clinicians may disagree about etiology and treatment, that is, but can at least join together in stamping out "mental disorder" as described in the generously broad *DSM *definitions. But this also is illusion, proved empirically to be so by the failure of every *DSM *definition to achieve widespread consensus and destined to be proved again in the inevitable failure of the *DSM-5 *definition. The reason for this is not that the definitions are poorly crafted (quite the opposite) but that such consensus, within the contemporary mental health landscape, is not a conceptual possibility. For example, with regard to the *DSM-5 *proposal - the best definition to date - particular clinicians are certain to reject not only nosological individualism but also the foundational assumptions behind "underlying psychobiological dysfunction," the exclusion of expectable responses to common stressors and losses, and the distinction between "behavioral" and "psychological." Furthermore, even if consensus on a formal definition were attainable, it would accomplish little since agreement about "dysfunction" and "impairment" and "deviance" can be no stronger than correlative agreement about proper human functioning in a particular situation (such that, e.g., what one clinician judges as impairment, another judges as unreasonable expectation). There is, unfortunately, no more agreement about proper human function than about the (closely related) nature of "mental health."

Crafting a definition of mental disorder is a useful thought-experiment for psychiatric nosologists and debating such definitions is great fun for the philosophically-minded. But in a document as influential and generally accessible as the *DSM*, such definitions elude and mislead more than they illumine. They should therefore be honorably retired.

### Commentary

Joseph Pierre, M.D.

UCLA Department of Psychiatry.

Developing an ironclad definition of mental illness (or for that matter the more general notion of disease) is indeed a daunting task [43,57-59]. Most attempts at a medical model definition are based on some variation on the concept of "something that has gone wrong biologically with an individual that results in distress or functional impairment," but problems with this approach quickly emerge. First, we have no firm explanations for what's biologically wrong in mental disorders [57] - no "underlying psychobiological dysfunctions" [43] have yet been elucidated (and as the joke goes, in the few cases where such "lesions" have been identified, they transform from psychiatric disorders into neurologic diseases). Second, the concepts of "wrongness" and "dysfunction" are unavoidably value-laden [57,60], while "distress" and "suffering" are subjective and relativistic [43,61]. Third, distinguishing between mental illness and "problems of living," "expectable responses to common stressors" (or extraordinary stressors), and "social deviance or conflicts with society" is at best challenging [43], given the inextricable, reciprocal relationship between individuals and their environments/cultures [58]. Therefore, in the absence of lab tests to detect biological lesions, psychiatric diagnosis inevitably rests upon some kind of "judgment call" on the part of a clinician and the inescapable conclusion that "no definition perfectly specifies precise boundaries for the concept of 'mental disorder [43].'"

As suggested earlier, clinicians do not in general fret over what does or does not constitute a disease. If, for example, a patient's arm is broken in a car accident, a doctor doesn't lose sleep pondering whether this represents "broken bone disorder" or simply an expected response to an environmental stressor - the bone is set and the arm is casted. For psychiatrists, ever since Freud's development of "The Talking Cure," the business of psychiatry has increasingly shifted from asylum care of patients with psychosis to outpatient treatment of the "worried well" [62]. Likewise, we now live in a society that regards the attainment of happiness as a worthwhile goal in life, if not an entitlement [63]. Therefore, mental disorder or not, clinicians working in "mental health" see it as their calling to try to improve the lives of whomever walks through their office door seeking help.

But clinical decisions are not the only decisions that hinge upon diagnosis. The underappreciated challenge to defining a mental disorder in the real world stems from the many different questions that are ultimately being asked of diagnosis:

Should "disorder X" be treated?

What is the best way to treat "disorder X"?

Should screening for "disorder X" be implemented in the community in the interest of treatment and prevention?

Should special school services be offered for children with "disorder X?"

Should insurance companies reimburse for care based on "disorder X?"

Should research funding be granted to study "disorder X?"

Should a patient population based on "disorder X" be selected for etiologic research?

Should someone with "disorder X" who has committed a crime be sentenced to prison or to involuntary psychiatric treatment?

The far-reaching implications of these questions render the distinction between mental illness and normality far more significant than when considered in a clinical vacuum, especially in the era of rationed healthcare services, insurance reimbursement, and competitive research funding. For the clinician and patient, erring on the side of assigning, or receiving, a diagnosis of a mental disorder has become incentivized, but future economic and policy decisions may necessitate a more conservative threshold for defining mental illness [61]. Therefore, the many questions asked of diagnosis cannot be answered by any single definition of mental illness, or by simply referring to the DSM. Instead, and in particular as DSM-5 embraces the concept of diagnostic spectra in which the borders between pathology and normality are stretched, wide-ranging consideration and context-specific analyses by clinicians, patients and their families, researchers, DSM architects, and policy-makers will be a vital, ongoing process that shapes the fate of modern psychiatry.

### Commentary

John Chardavoyne, M.D.

Yale University Department of Psychiatry.

How should American psychiatry define "mental disorder?" There is a varied array of dimensions that are emphasized in the various definitions, including the biological basis of psychiatric illness; the behavioral manifestations; the severity of impairment; the level of distress; and the differentiation between pathology versus normalcy. In this commentary I will provide examples of these different aspects, suggest reasons for the confusion, and propose the beginning of a more integrated approach.

First, let's review elements of a few of the definitions that have been proposed. The DSM-IV definition emphasizes explicitly that it characterizes disorders, not people; that there are biological, psychological, or behavioral dysfunctions that result in a psychological or behavioral syndrome; this creates impairment or distress; it is not a result from discord between the individual and society; and that these problems are not culturally-sanctioned. http://www.DSM5.org/ProposedRevisions/Pages/proposedrevision.aspx?rid=465#. The proposed changes for DSM-V include that there is psychobiological dysfunction that results in a psychological or behavioral syndrome; that there is evidence of distress or impairment in functioning; that the response is not expectable and not culturally-sanctioned; it is not a result from discord between the individual and society; and that there is diagnostic validity and utility. http://www.DSM5.org/ProposedRevisions/Pages/proposedrevision.aspx?rid=465. According to the National Alliance on Mental Illness, mental illness is defined with an emphasis on the medical model. http://www.nami.org/Content/NavigationMenu/Inform_Yourself/About_Mental_Illness/About_Mental_Illness.htm. The International Classification of Diseases-10 definition highlights the symptoms. http://www.who.int/classifications/icd/en/bluebook.pdf.

These definitions reflect the difficulties in adequately describing the nature of a "mental disorder." They reflect the uncertainty about how biology results in psychiatric manifestations and vice versa and difficulties in the following: the way to differentiate pathology from normalcy, how to categorize the syndromes, and the location of the source of dysfunction. Along these lines, a chief concern is the emphasis on overt behaviors to classify mental disorders. Despite how patients report subjective experiences and distress, the overt behaviors have been used as the major indicators of disorder. The difficulties related to quantifying the first-person subjective experience compared to the third-person objective observations (overt behaviors) manifest here. The Psychodynamic Diagnostic Manual reintroduces the subjective experience into assessment [64].

This begs the question of the direction that psychiatry wants to go. Do psychiatrists want to be able to treat an individual with manic symptoms and also someone with intimacy problems? Presumably the former would have more behavioral markers than the latter. However, how can one quantify the suffering of one versus the other? Simply because there are not as many behavioral manifestations, does that mean that the person is not suffering sufficiently to warrant treatment by a psychiatrist? Equally important, does that suggest that insurance companies continue to determine reimbursement for treatment based on behaviors without considering level of suffering and other factors that contribute towards psychological dysfunction like thoughts, feelings, and relationships?

Another element to consider when defining "mental disorder" is the fact that there is a person foremost who has the signs and symptoms of a mental disorder. As referenced earlier, the DSM-IV states that the definition focuses on the disorder and not the person. http://www.DSM5.org/ProposedRevisions/Pages/proposedrevision.aspx?rid=465#. This portends various problems because psychiatrists treat people. This fact can be lost in this age of fifteen-minute medication checks and insurance pressures. The essential aspect of the help is the relationship so acknowledgement of the patient as a person with an illness should be added to the definition of mental disorder. "... When therapists apply manualized treatments to selected symptom clusters without addressing the complex person who experiences the symptoms and without attending to the therapeutic relationship that supports the treatment, therapeutic results are short-lived and rates of remission are high." [64]Admittedly, what complicates this issue is our lack of understanding of how brain processes (ion fluxes, neurotransmission, etc) result in consciousness, intentionality, thoughts, and the subjective experience of emotions and how these psychological states affect brain processes. Along this vain, perhaps the use of "mental" should be reconsidered because it implies, intentionally or not, the separation of mind and brain. Perhaps "neuro-mental" could be considered? The definition possibly could begin like this: "An individual (with hopes, dreams, disappointments, and feelings like his treaters) is considered to have a neuro-mental disorder when...."

The definition of a mental disorder will evolve over time as our knowledge advances and cultures evolve. It may be worthwhile to recognize that a definition will be merely a synthesis of the various dialectical poles and will require repeated adjustments over time. If we're willing to change our understanding of the definition, then perhaps we'll be willing to better understand our patients.

### Commentary: The Difficulties of Defining a Mental Disorder in DSM-III

Hannah S. Decker, Ph.D.

University of Houston.

I am commenting on this question as a historian of psychiatry who is writing a book on the making of DSM-III.

The first two DSMs in 1952 and 1968 eschewed a definition of mental disorder befitting their modest origins from nomenclatures that served primarily "the [statistical] needs and case loads of public mental hospitals" [[65], p. vi]. But the editor of the third edition (1980), Robert L. Spitzer, had more ambitious goals for the manual resulting in a volume that was over three times the size of DSM-II and had dozens of new diagnoses. It was Spitzer's plan to include in DSM-III a definition of "mental illness" as a subset of "medical illness." Circumstances forced him to abandon this type of definition, but there is a definition of "mental disorder" in DSM-III, prefaced by the familiar caveat, "there is no satisfactory definition that specifies precise boundaries for the concept 'mental disorder'" [[66], p. 5].

Spitzer wanted to establish that, without any doubt, psychiatry was a part of medicine. He had initially thought seriously about mental disorders even before he was appointed the head of the Task Force. In 1973, he had brokered the removal of the diagnosis of homosexuality as a mental disorder from DSM-II, and the controversy surrounding the event sensitized him to the subject of what constituted a mental disorder. He soon found impediments to his goal of establishing definitions of medical and mental disorder in DSM-III. Still, at every turn he persevered because he envisioned the issuance of the new diagnostic manual as having intellectual goals far larger than its being a diagnostic classification. Spitzer wanted DSM-III to play a role in combating the anti-psychiatry movement of the 1960s and early '70s and to refute critics such as Thomas Szasz who said mental illness was a myth.

I would like to spell out briefly the obstacles that lay in the path of an agreed-upon definition of a mental disorder. At the annual meeting of the American Psychiatric Association in May 1976, Spitzer and Jean Endicott, a close colleague on the DSM-III Task Force, put forth their definitions of medical and mental disorders. The reaction was quite negative. As Spitzer later reported: "Some questioned the need and wisdom of having any definition. Many argued that the definition proposed was too restrictive, and if officially adopted, would have the potential for limiting the appropriate activities of our profession... they also felt that it was out of keeping with trends in medicine that emphasize the continuity of health and illness" [[67], p. 16]. (This continues to be an important question in current debates over what diagnoses should be in DSM-5. Allen Frances, in particular, has argued against pathologizing what he sees as aspects of normality, "everyday incapacity," in his words.)

Then, Spitzer encountered strenuous opposition from psychologists to the notion that mental disorders were medical disorders. This was a turf issue, with the psychologists fearing that they would lose the right to treat mental disorders if they were defined as medical. In June 1976, a conference was held in St. Louis on "Critically Examining DSM-III in Midstream." Dr. Maurice Lorr, representing the American Psychological Association, "expressed the view that mental disorders (as medical disorders) should be limited to those conditions for which a biological etiology or pathophysiology could be demonstrated." In addition, just two months earlier, a former president of the American Psychological Association had been quite blunt in expressing his view that DSM-III was "turning every human problem into a disease, in anticipation of the shower of health plan gold that is over the horizon" [[67], p. 36].

In spite of these disagreements, Spitzer, as was his wont, did not surrender easily. He returned the next year to bolster his arguments. This was at the yearly meeting of the American Psychopathological Association, an organization of preeminent American psychiatrists dedicated to research on human behavior. In 1977 it devoted its annual conference to "Critical Issues in Psychiatric Diagnosis." Spitzer and Endicott not only presented retooled definitions of both medical and mental disorders, but Spitzer, as an editor of the 1978 published proceedings of the conference, now took the opportunity to remind his readers of the blows psychiatry had endured in the 1960s and early '70s: "The very concept of psychiatric illness has been under considerable attack in recent years. This attack has largely depended upon studies derived from the social sciences. Some have taken the stand that what are called mental illnesses are simply those particular groups of behaviors that certain societies have considered deviant and reprehensible." Spitzer believed that this rejection of the legitimacy of psychiatry was partly owed to the fact that "no generally agreed upon definition of mental illness has been propounded that is not open to the criticisms of cultural relativism" [[68], p. 5].

In addition to his conviction that DSM-III, with its new diagnostic criteria, would bring diagnostic reliability to psychiatry, Spitzer conceived of the new DSM as a weapon that could repel psychiatry's cultural challengers. The new manual would thus have a potential of historical proportions. Nevertheless, although Spitzer labored mightily to develop "mental illness" as a subset of "medical illness," he was ultimately forced to bow both to the opinions of his psychiatric colleagues, who had philosophical and practical objections to his definition, and to the demands of the psychologists that mental illnesses be labeled "mental disorders." The upshot was that mental disorders did not get to be defined as medical disorders.

The attempts of Robert Spitzer--a psychiatrist of considerable accomplishment in many areas of the profession--to establish a definition of a mental disorder, illustrate the complexities of arriving at one that is intellectually satisfying, clinically useful, and practically acceptable. Nevertheless, he did include a definition of mental disorder in DSM-III under the category of "Basic Concepts" [[66], pp. 5-6]. DSM-IV [[69], pp. xxi-xxii], with some changes, essentially preserved Spitzer's definition, which also forms the basis of the definition planned for DSM-5 [70] (See also Stein et al [43]. The individuals addressing this latest revision include the habitual warning: "No definition perfectly specifies precise boundaries for the concept of either 'medical disorder' or 'mental/psychiatric disorder'" [70].

### Allen Frances responds: Mental Disorder Defies Definition

Humpty Dumpty: "When I choose a word it means just what I choose it to mean"

When it comes to defining the term "mental disorder" or figuring out which conditions qualify, we enter Humpty's world of shifting, ambiguous, and idiosyncratic word usages. This is a fundamental weakness of the whole field of mental health.

Many crucial problems would be much less problematic if only it were possible to frame an operational definition of mental disorder that really worked. Nosologists could use it to guide decisions on which aspects of human distress and malfunction should be considered psychiatric- and which should not. Clinicians could use it when deciding whether to diagnose and treat a patient on the border with normality. A meaningful definition would clear up the great confusion in the legal system where matters of great consequence often rest on whether a mental disorder is present or absent.

Alas, I have read dozens of definitions of mental disorder (and helped to write one) and I can't say that any have the slightest value whatever. Historically, conditions have become mental disorders by accretion and practical necessity, not because they met some independent set of operationalized definitional criteria. Indeed, the concept of mental disorder is so amorphous, protean, and heterogeneous that it inherently defies definition. This is a hole at the center of psychiatric classification

And the specific mental disorders certainly constitute a hodge podge. Some describe short term states, others lifelong personality. Some reflect inner misery, others bad behavior. Some represent problems rarely or never seen in normals, others are just slight accentuations of the everyday. Some reflect too little control, others too much. Some are quite intrinsic to the individual, others are defined against varying and changing cultural mores and stressors. Some begin in infancy, others in old age. Some affect primarily thought, others emotions, yet others behaviors, others interpersonal relations, and there are complex combinations of all of these. Some seem more biological, others more psychological or social.

If there is a common theme it is distress and disability, but these are very imprecise and nonspecific markers on which to hang a definition. Ironically, the one definition of mental disorder that does have great and abiding practical meaning is never given formal status because it is tautological and potentially highly self serving. It would go something like "Mental disorder is what clinicians treat and researchers research and educators teach and insurance companies pay for." In effect, this is historically how the individual mental disorders made their way into the system.

The definition of mental disorder has been elastic and follows practice rather than guides it. The greater the number of mental health clinicians, the greater the number of life conditions that work their way into becoming disorders. There were only six disorders listed in the initial census of mental patients in the mid nineteenth century, now there are close to three hundred. Society also has a seemingly insatiable capacity (even hunger) to accept and endorse newly defined mental disorders that help to define and explain away its emerging concerns.

As a result, psychiatry is subject to recurring diagnostic fads. Were DSM5 to have its way we would have a wholesale medicalization of everyday incapacity (mild memory loss with aging); distress (grief, mixed anxiety depression); defects in self control (binge eating); eccentricity (psychotic risk); irresponsibility(hypersexuality); and even criminality (rape, statutory rape). Remarkably, none of these newly proposed diagnoses even remotely pass the standard loose definition of "what clinician's treat". None of these "mental disorders" has an established treatment with proven efficacy. Each is so early in development as to be no more than "what researchers research" - a concoction of highly specialized research interests.

We must accept that our diagnostic classification is the result of historical accretion and accident without any real underlying system or scientific necessity. The rules for entry have varied over time and have rarely been very rigorous. Our mental disorders are no more than fallible social constructs.

Despite all these limitations, the definitions of mental disorders contained in the DSM's are necessary and do achieve great practical utility. The DSM provides a common language for clinicians, a tool for researchers, and a bridge across the clinical/research interface. It offers a textbook of information for educators and students. It contains the coding system for statistical, insurance, and administrative purposes. DSM diagnoses also often play an important role in both civil and criminal legal proceedings. The DSM system is imperfect, but indispensable.

It is undoubtedly a failing on my part, but I find myself unable to take much interest in efforts to define mental disorder. My too practical temperament prefers to spend my too limited time on earth attending to concrete and soluble problems and studiously avoids the abstract and the insoluble. Defining mental disorder in a useful way clearly lies above my intellectual pay grade.

This is not to say that the question is uninteresting or unimportant. Would that there were a workable definition of mental disorder. We could then comfortably decide which of the proposed mental disorders need be included in the DSM, which aspects of human suffering and deviance are best left out. We could also come to a ready judgment about each individual potential 'patient'- who best qualifies for diagnosis and treatment, who is best left to his own devices.

Alas, however, the sheep and the goats refuse to declare themselves in any convenient and discernible way. The definitions of mental disorder offered here make perfect sense in the abstract, but provide no guidance on how to make concrete decisions. They do not tell us, for example, whether mixed anxiety depression or binge eating or the early forgetting of advanced years are disorders or facts of life. They do not guide us in diagnosing the many people who populate the fuzzy boundary between mental disorder and normality.

Seeing no practical consequence, I have no opinion on the fine points of definition- since these seem to be of only academic interest. Mental disorder is (like 'disease' and 'obscenity' or 'love') something you hope you can spot when you see it, but by implicit rules that inherently are poorly defined and ever shifting.

#### Reply to Dr Wakefield

If anyone in the world could usefully define mental disorder, it would be Jerry Wakefield. He has tried long, hard, skillfully, even brilliantly and has come up with a definition that works extremely well on paper. His "harmful dysfunction" and evolutionary perspective provide the best possible abstract definition of mental disorder. The problem is that Dr Wakefield's definition is not operational in a way that provides guidance on the two questions that most count: 1) Is this proposed new diagnosis a mental disorder that should be included in the official nomenclature? 2) Does this person have sufficient psychiatric problems to warrant a diagnosis of mental disorder? Unfortunately, neither question lends itself to his definitional solution. As Dr Wakefield himself points out, "both the notions of dysfunction and harm are fuzzy concepts" that are only useful to "determine a range of clear cases on either side of the disorder/nondisorder boundary." We are left to settle the crucial and frequent tough fuzzy boundary questions in what remains a necessarily unsatisfactory, ad hoc, and often idiosyncratic manner.

Dr Wakefield seems to accept the necessity of my less abstract "cost/benefit analysis" approach for reducing the reckless DSM 5 diagnostic exuberance at the fuzzy boundary with normality. But he goes on rightly to criticize its susceptibility to misuse in the service of social control or economic manipulation. These issues are taken up in more detail in question #4 on the limitations of pragmatics in framing the diagnostic system.

Dr Wakefield and I agree completely on the most important question facing psychiatry today- the risk of false positives and excessive treatment. Diagnostic inflation has been a huge problem in the way DSM IV has been used. It will be greatly amplified by the many new high prevalence diagnoses being suggested for DSM 5. Unfortunately, no available definition of mental disorder has been able to withstand the pressure of lowered diagnostic standards and drug company advertising.

#### Response to Dr Kinghorn

I agree that the DSM's gain little from attempting to provide an abstract definition of mental disorder and that having a useless definition may be worse than offering no definition at all. Also, let's remember that we are not alone in being definitionally challenged- there is really no good operational definition in medicine for "disease" or "illness."

#### Response to Dr Pierre

I agree completely with Dr Pierre's eloquent critique of the definitions of mental disorder and fully endorse his concern that such consequential decisions rest on such a fragile tissue. It will be important to get the widest input on all the crucial questions raised by Dr Pierre. We can't rely on any definition of mental disorder, nor can we trust the wisdom of experts on that disorder or of any single professional association. The decisions about what constitutes a mental disorder require the same safety care as the FDA devotes before allowing the introduction of a new drug.

#### Reply to Dr Chardavoyne

I understand Dr Chardavoyne's regret that DSM may seem to lose the person in its effort to define the disorder. I just don't see a solution within the diagnostic system- which necessarily has to focus on symptom similarities, rather than the particularities and idiosyncrasies that make each of us who we are. Holding fast to the person is the crucial task of every clinician, but it is not something the DSM can help with.

#### Reply to Dr Decker

Hannah Decker does us a great service by recalling and recording attempts to define mental disorder. I am afraid, however, that this is a situation in which knowing a problematic past is insufficient to avoid repeating it. There will always be a strong desire to define 'mental disorder' because it is so important in setting the boundary with normality. But all efforts at universal definition will fail because the concept is so inherently fuzzy and situation bound. The only consolation is that 'medical illness' is equally vague and hard to define.

## Conclusion

The two questions covered in this article form a natural pair. How you define mental disorder (Question 2) will certainly depend on what you think mental disorders are and how we are able to know about them (Question 1). I will briefly summarize the discussion developed in these questions and save a larger review for the general conclusion.

As indicated above in the General Introduction, the startling failure of research to validate the DSM categories of DSM-III and DSM-IV has led to a conceptual crisis in our nosology: what exactly is the status of DSM diagnoses? Do they identify real diseases, or are they merely convenient (and at time arbitrary) ways of grouping psychiatric symptoms? These are the issues dealt with in Question 1, framed in the umpire metaphor introduced by Allen Frances in his "DSM in Philosophyland" piece published in Bulletin 1 and commented on at length in Bulletin 2. The commentaries in this article roughly follow the positions of the five imagined umpires, although, as explained above, most of us will not restrict ourselves to a purist version of one of the umpires. Indeed, Frances himself, while stating a clear point of view, acknowledges that each umpire position captures a bit of the truth.

The first two commentaries address Question 1 in a broad way, commenting on the process of deciding about the merits of the various positions. Peter Zachar and Stephen Lobello are scholars who take their baseball seriously and weave the metaphor into a complex analysis in which their pragmatic (practical kinds) perspective subsumes all the umpires, including the pragmatic fourth umpire. In her commentary Claire Pouncey doesn't quite assume a position but provides a clear presentation of the differences among the first three umpires. She begins with the clarification that the umpire question involves both ontology and epistemology: what is there, and can I know it. Umpire 1 is a Strong Realist - both an ontological realist and an epistemological realist. Umpire 2 is a Strong Realist/Weak Constructivist - an ontological realist and an epistemological less-than-realist. Umpire 3 is a Strong Constructivist - an ontological anti-realist and an epistemological anti-realist.

In his commentary Nassir Ghaemi offers a spirited defense of a realist, first-umpire position, challenging those who don't accept the reality of mental illnesses as to what they're doing treating patients. I am calling him a first umpire, but he rejects the umpire metaphor, offering in its stead Kenneth Kendler's notion of "epistemic iteration."

The next three commentators assume some variation on the "nominalist" second umpire position. Michael Cerullo invokes the naturalist/normativist debate, a distinction that echoes Jerome Wakefield's harmful dysfunction notion of psychiatric disorders. Cerullo argues that all diseases, including psychiatric disorders, have natural and normativist aspects, although some lean more toward the naturalist dimension and others toward the normativist dimension.

In his contribution Jerome Wakefield follows with a thorough presentation of his well-known harmful dysfunction understanding of mental disorders. For purposes of the umpire discussion he locates his HD umpire in a humble realist 1.5 position - nominalism with a tilt toward realism.

Finally, Joseph Pierre invokes the fate of the planet Pluto to point to the reality of things studied by science and reminds us of the biological reality of mental disorders; but, acknowledging the uncertainties of our knowledge, he assumes the second umpire position. Like the others in the second umpire group, he notes that some psychiatric disorders make more claim on a first umpire stance than others.

Gary Greenberg boldly assumes the third umpire position, even invoking Samuel Johnson's kick in a face-off with Ghaemi's use of the kick to defend the first umpire. Greenberg argues that the human interest is so powerful in determining what counts as disease and what does not that honesty drives us to the constructivist stance.

In his commentary Harold Pincus elaborates the very diverse ways in which concepts of mental disorders are used by an assortment of user groups, leading him to emphasize the fourth umpire, pragmatist, position toward psychiatric conditions. He argues cogently that validity as we now know it will not be a meaningful concept in the future.

Finally, with his usual energy and without any indication of retreat, Thomas Szasz comfortably assumes the position of fifth umpire and reviews the stance toward psychiatric disorders he familiarized us with fifty years ago.

And still finally, in a reflection that probably belongs best with the fifth umpire, Elliott Martin argues that the insurance industry has so co-opted the nosology that we might consider it the only umpire in the game.

In his response to the commentaries on Question 1, Allen Frances begins by noting that "[n]one of the five umpires is completely right all of the time. And none is totally wrong all of the time. Each has a season and appropriate time at the plate." He then proceeds to a historical perspective, noting that in the heyday of biological psychiatry forty years ago, Umpire 1, 3, and 5 were ascendant. On the one hand, the biological psychiatrists were confident that the realist position of Umpire 1 would prevail. And on the other hand, they were challenged by a broad range of skeptics occupying the positions of the Umpires 3 and 5. In Frances' account that has all changed. Chastened by the failures of biological psychiatric to produce, but convinced of the reality of psychiatric illness, we as a majority have gravitated toward the position of Umpire 2 - there is certainly psychiatric illness, but the categories of DSM-III and IV may not have carved those infamous joints correctly. Frances offers a guarded defense of the categories, nonetheless, arguing that, until further science has settled the issue of what are valid categories, the current ones serve a useful function of organizing the clinical phenomena which we confront in our work. The pragmatic Umpire 4 thus has a say in our current efforts to diagnose. "Mental disorders are no more and no less than constructs. And Umpire 4 is quick to point out that they are very useful constructs." Frances ends on an optimistic note that with more scientific clarity in the future, we can anticipate that Umpire 1 will gradually assume prominence over Umpire 2.

With the second question, definition, Frances can certainly claim more experience that most of us because of the time he put in grappling with this question in DSM-IV. His dissatisfaction with his own work product, and his skepticism about ever getting it right are certainly revealing - and consistent with his response to the first question. He points to the heterogeneity of what gets called a mental disorder, as well as to the unavoidable fact that many have been admitted to the club through the distinctly unscientific process of historical accretion. All that said, he argues that the DSM categories serve a very important role in facilitating communication among mental health professional and are thus necessary, however imperfect and imprecise. He concludes on a note of flagging interest in settling this question.

The commentators have stretched themselves to the imaginable extremes in tackling this question. The majority, along with Frances, view the DSM as a very motley assortment of behaviors and states of mind, and they see the DSM definition as trying to accommodate what we in fact treat, as opposed to leading us to decide what we *should *treat. The exception is Jerome Wakefield, who has argued persuasively for some time - and rehearses the main features of his argument here - that we can provide a clear of definition of mental disorder with the notion of harmful dysfunction. This is a definition that covers both the scientific and normative aspects of mental disorder, and that purports to guide us rather than follow us in our practice.

The commentaries at the other end of the spectrum start with Warren Kinghorn, who argues that since the DSM definition accomplishes nothing, even what it minimally claims to accomplish - organizing the terrain and establishing common goals of practice - we should acknowledge that we don't use it, don't need it, and should just retire it.

Joseph Pierre offers another argument for the impossibility of developing an adequate definition - poor science, value intrusion, ever broadening parameters of practice - and also reminds us that general medicine does quite well without an official definition. In spite of his cogent argument for the failed project of definition, he appears to stop short of Kinghorn's recommendation, and without mustering Frances' defense, appears to be in favor of having the definition, however inadequate. That is presumably because, unlike, Kinghorn, he feels that an official definition can have significant consequences for the field of psychiatry.

John Chardavoyne, makes a plea for escaping the inadequate science by reorienting the definition away from the disease and back to the person. In doing this he retains a definition but stands somewhat outside the debate that has engaged Frances and the other commentators.

In a final commentary Hannah Decker takes a look back and reviews Robert Spitzer's struggles to develop a definition of mental illness for DSM-III - a commentary that prompts Allen Frances to remark that this is a situation in which even a thorough examination of the past may not improve our performance in the present.

## Competing interests

MF is an external consultant to the NIMH Research Domain Criteria (RDoC) Project. NG has research grants from Pfizer and Sunovion, and is a research consultant for Sunovion. MS is a consultant for AstraZeneca, Merck, Novartis and Sunovion. Other authors report no competing interests.

## Authors' contributions

JP(Phillips) wrote the general General Introduction and Conclusion, as well as the introductions to the individual conclusions. AF wrote the Responses to Commentaries. MC, JC, HD, MF, NG, GG, AH, WK, SL, EM, AM, JP(Paris), RP, HP, DP, CP, MS, TS, JW, SW, OW, PZ wrote the commentaries. All authors read and approved the final manuscript.
